# HER2-Low and HER2-Ultralow Metastatic Breast Cancer and Trastuzumab Deruxtecan: Common Clinical Questions and Answers

**DOI:** 10.3390/cancers17244021

**Published:** 2025-12-17

**Authors:** Nusayba A. Bagegni, Karthik V. Giridhar, Daphne Stewart

**Affiliations:** 1 Division of Oncology, School of Medicine, Washington University in St. Louis, St. Louis, MO 63110, USA; 2 Department of Oncology, Mayo Clinic, Rochester, MN 55905, USA; 3 USC Norris Comprehensive Cancer Center, Los Angeles, CA 90033, USA

**Keywords:** breast cancer, precision medicine, T-DXd, ADC, HER2-low, HER2-ultralow

## Abstract

Approximately 80% of invasive breast cancers are classified as human epidermal growth factor receptor 2 (HER2)-negative; however, many of these tumors have detectable levels of HER2 surface expression. Trastuzumab deruxtecan (T-DXd) is a HER2-directed antibody-drug conjugate with a membrane-permeable payload that is cytotoxic to both HER2-expressing tumor cells and neighboring cells via the bystander antitumor effect. T-DXd has shown significant antitumor activity in clinical trials for patients with HER2-positive (immunohistochemistry [IHC] 3+ or IHC 2+/in situ hybridization [ISH]+) breast cancer. In addition, the results of the DESTINY-Breast04 trial demonstrated the clinical benefit of T-DXd in patients with HER2-low (IHC 1+ or IHC 2+/ISH−) breast cancer after receiving prior chemotherapy. DESTINY-Breast06 demonstrated the clinical benefit of T-DXd in patients with hormone receptor (HR)-positive, HER2-low (IHC 1+ or IHC 2+/ISH−), and HER2-ultralow (IHC 0 with membrane staining) metastatic breast cancer who had not received prior chemotherapy in the advanced setting. These results validate the need for a standard-of-care diagnostic test to identify HER2-low and HER2-ultralow expression levels in patients with metastatic breast cancer to guide therapeutic decision-making. Furthermore, effective treatment sequencing strategies and adverse event management are essential for maximizing patient benefit. This review presents the identification of HER2-low and HER2-ultralow breast cancer, sequencing of T-DXd with other treatments, and management of common or clinically significant adverse events reported with T-DXd.

## Simple Summary

Historically, cases of breast cancer were categorized as either human epidermal growth factor receptor 2 (HER2)-negative or HER2-positive. However, many cases classified as HER2-negative express detectable levels of the HER2 protein and can be described as HER2-low (immunohistochemistry [IHC] 1+ or IHC 2+/in situ hybridization [ISH]+) or HER2-ultralow (IHC 0 with membrane staining). Trastuzumab deruxtecan (T-DXd), a HER2-directed antibody linked to a chemotherapy compound, has shown clinical benefits in HER2-positive breast cancer. Further, it has demonstrated clinical activity in HER2-low and HER2-ultralow breast cancers, opening the door for HER2-directed therapies as a new treatment option for these patients. This review highlights the importance of HER2 testing and classification to help guide treatment decisions. We also discuss T-DXd treatment outcomes, adverse event management, and the sequence of available treatments for patients with HER2-low and HER2-ultralow advanced breast cancer.

## 1. Introduction

Breast cancer can be characterized by tumor heterogeneity with diverse molecular subtypes that require tailored treatment strategies [1]. Therapeutic decisions are guided by hormone receptor (HR) status (estrogen receptor [ER] and/or progesterone receptor [PR]) and human epidermal growth factor receptor 2 (HER2) status [1].

**Figure 1:**
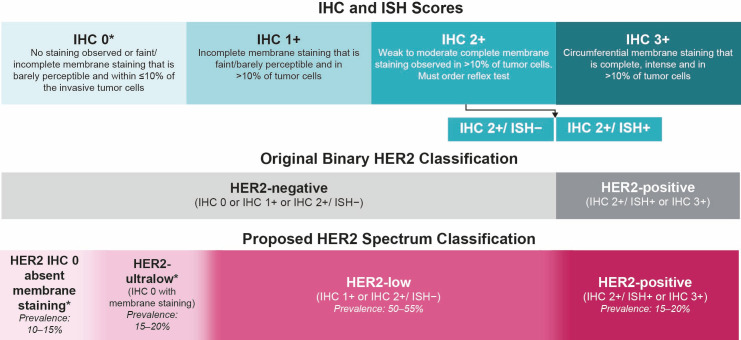
Current and suggested HER2 classification [2,3,5,11,12,13,14,15,16,17]. HER2, human epidermal growth factor receptor 2; IHC, immunohistochemistry; ISH, in situ hybridization. * The subdivision of IHC 0 into two categories has been proposed: HER2-ultralow (incomplete or faint/barely perceptible membrane staining in ≤10% of the invasive tumor cells) and HER2 IHC 0 absent membrane staining [5].

HER2 testing has historically been used to identify HER2-pathway activation, which predicts responsiveness to established HER2-targeted therapies such as trastuzumab [2]. HER2 status is assessed through immunohistochemistry (IHC) and in situ hybridization (ISH) testing of tumor samples (Figure 1).

According to the 2018 American Society of Clinical Oncology–College of American Pathologists (ASCO–CAP) clinical practice guidelines on HER2 testing in breast cancer, tumors are classified as HER2-positive (defined as IHC 3+ or IHC 2+/ISH+) or HER2-negative (defined as IHC 0, IHC 1+, or IHC 2+/ISH−; Figure 1) [3]. However, HER2 expression exists on a spectrum, and most tumors classified as HER2-negative express detectable amounts of the HER2 protein [4,5]. Recent literature has introduced more granular categories for HER2-negative breast cancer: HER2-low (IHC 1+ or IHC 2+/ISH−), HER2-ultralow (IHC 0 with membrane staining), and HER2 IHC 0 absent membrane staining (Figure 1) [5,6,7,8].

Although neither HER2-low nor HER2-ultralow has been formally recognized as categories of HER2 expression per ASCO–CAP guidelines, the HER2-directed antibody–drug conjugate (ADC) trastuzumab deruxtecan (T-DXd) has demonstrated clinical activity in tumors with these expression levels [6,7,8]. Results from DESTINY-Breast04 prompted the 2023 ASCO–CAP guidelines update, which now states that it is best practice to distinguish IHC 0 from 1+ in pathology reports [2,6,9]. Furthermore, based on the results from the DESTINY-Breast06 trial, T-DXd is now approved for the treatment of patients with HR-positive, HER2-low, or HER2-ultralow advanced breast cancer, as determined by a locally or regionally approved test, that has progressed on one or more endocrine therapies in the metastatic setting [10].

In this narrative review, we discuss the importance of testing and identifying patients with HER2-low and HER2-ultralow breast cancer. We summarize the treatment outcomes of T-DXd in the DESTINY-Breast04 and DESTINY-Breast06 trials, and we explore the sequence of available treatments for patients with HER2-low and HER2-ultralow advanced breast cancer. We also discuss the management of common and clinically significant adverse events reported with T-DXd.

### What Is the Prevalence and Unmet Need of the Patient Population with HER2-Low and HER2-Ultralow Breast Cancer?

Approximately 80% of invasive breast cancers are categorized as HER2-negative, as per ASCO–CAP guidelines [2,3,14]; however, across studies of patients with primary or metastatic breast cancer, an estimated 50–55% meet the criteria for HER2-low and 15–20% meet the criteria for HER2-ultralow breast cancer (Figure 1) [13,14,15,16,17]. Currently, HER2-low and HER2 IHC 0 tumors do not appear to be distinct biological subtypes of breast cancer [3,5,18,19,20]. Approximately 88% of HER2-low breast cancer cases are HR-positive and generally have a better prognosis than those with HR-negative, HER2-low tumors [21,22,23]. Treatment options are limited for both subgroups due to inevitable resistance to endocrine therapy (ET) and chemotherapy [24,25,26]. For advanced HR-positive, HER2-negative breast cancer, first-line treatment typically consists of ET combined with a cyclin-dependent kinase 4/6 inhibitor (CDK4/6i) [27,28,29,30,31,32]. Upon disease progression, second-line treatment may include a different ET, either alone or in combination with a targeted therapy or chemotherapy [27,28,29,30,31,32]. Rechallenge with ET can delay the initiation of chemotherapy; however, the eventual development of ET resistance is common and remains a challenge for long-term disease control [30]. Treatment efficacy decreases with disease progression, as reflected in clinical trial results showing a median progression-free survival (PFS) of 20.2–30.6 months with first-line ET plus CDK4/6i therapy compared with 4.2–8.1 months with second-line ET regimens [33,34,35,36,37,38]. In addition to diminishing efficacy, treatment discontinuation reduces the proportion of patients who receive subsequent lines of therapy (patient attrition rates), which among patients with HR-positive, HER2-negative breast cancer progressing from the first to the second line of therapy has been reported to be 15–33% in clinical trials and 18–51% in real-world evidence studies, primarily due to disease progression and death [33,39,40,41,42,43,44,45,46]. The combination of reduced efficacy and high attrition rates emphasizes an unmet need for improved therapeutic options for patients with HER2-low, HER2-ultralow, and HER2 IHC 0 absent membrane staining metastatic breast cancer that has progressed following standard treatments [25,46].

For patients with advanced HR-negative, HER2-negative breast cancer, also known as triple-negative breast cancer (TNBC), standard first-line treatment includes chemotherapy, immunotherapy with chemotherapy (for patients with programmed death ligand 1 [PD-L1]–positive tumors), or poly (ADP-ribose) polymerase inhibitors (PARPi; for patients with germline *BRCA1*/*2* mutations) [26,27,28,31,32]. Historically, the duration of response to chemotherapy has been limited by progressive chemoresistance [26]. Clinical trials of chemotherapy with or without immunotherapy have shown a median PFS ranging from 1.5 to 10.9 months in the first- and second-line settings [47,48,49,50].

## 2. T-DXd Efficacy in HER2-Low and HER2-Ultralow Breast Cancer

### 2.1. How Does T-DXd Target HER2-Low and HER2-Ultralow Breast Cancer, and How Effective Is T-DXd in These Tumors?

T-DXd is a HER2-directed ADC composed of a humanized anti-HER2 antibody bound to a topoisomerase I inhibitor payload via an enzymatically cleavable tetrapeptide-based linker [51,52,53]. In the proposed mechanism of action, the membrane-permeable T-DXd payload can destroy targeted tumor cells expressing HER2 and diffuse into neighboring cells, regardless of HER2 status; this is known as the bystander antitumor effect [51,52,53,54]. In addition, a nonclinical study has indicated that the presence of extracellular proteases, such as cathepsin L, within the tumor microenvironment may contribute to the activity of T-DXd in HER2-low and HER2-ultralow tumors [55]. Clinical activity of T-DXd in patients with HER2-low and HER2-ultralow tumors has been reported in the DESTINY-Breast04 (NCT03734029) and DESTINY-Breast06 (NCT04494425) trials [6,8].

### 2.2. Evidence for T-DXd in HR-Positive, HER2-Low Breast Cancer

DESTINY-Breast04 was a randomized, open-label, phase 3 trial designed to investigate the efficacy and safety of T-DXd (5.4 mg/kg) compared with treatment of physician’s choice (TPC; which included capecitabine, eribulin, gemcitabine, paclitaxel, or nab-paclitaxel) in patients with HR-positive or HR-negative, HER2-low, unresectable or metastatic breast cancer who had received one or two prior lines of chemotherapy [6]. At data cutoff (DCO) for the primary analysis (11 January 2022), the median PFS by blinded independent central review (BICR) in patients in the HR-positive cohort was 10.1 months with T-DXd (*n* = 331) versus 5.4 months with TPC (*n* = 163; hazard ratio, 0.51; *p* < 0.001); median overall survival (OS) in this cohort was 23.9 months with T-DXd versus 17.5 months with TPC (hazard ratio, 0.64; *p* = 0.003; Table 1) [6]. The results of this trial demonstrated that T-DXd had statistically significant and clinically meaningful activity with a consistent safety profile in patients with HR-positive, HER2-low metastatic breast cancer. These findings established HER2-low as a clinically relevant breast cancer subtype that impacts future treatment for patients [6].

While DESTINY-Breast04 evaluated T-DXd after one or two prior line(s) of chemotherapy in HER2-low advanced disease, DESTINY-Breast06, a randomized, open-label, phase 3 study, compared earlier introduction of T-DXd versus TPC (capecitabine, nab-paclitaxel, or paclitaxel) in patients with HR-positive, HER2-low, or HER2-ultralow metastatic breast cancer whose disease progressed on ET in the metastatic setting and who had received no prior lines of chemotherapy [8]. At the first interim analysis DCO (18 March 2024), T-DXd achieved a statistically significant and clinically meaningful improvement in the primary endpoint, PFS by BICR, compared with TPC. Median PFS in the HER2-low cohort was 13.2 months with T-DXd versus 8.1 months with TPC (hazard ratio, 0.62; *p* < 0.001; Table 1) [8]. OS data were not mature at this DCO (39.6% maturity); the 12-month OS survival rate was 87.6% with T-DXd and 81.7% with TPC [8].

### 2.3. Evidence for T-DXd in HR-Negative, HER2-Low Breast Cancer

The DESTINY-Breast04 trial included an exploratory HR-negative, HER2-low cohort of 58 patients (T-DXd, *n* = 40; TPC, *n* = 18) [6]. When treated with T-DXd compared with TPC, patients within this population had an improved confirmed objective response rate (ORR; 50.0% vs. 16.7%), median PFS (8.5 vs. 2.9 months; hazard ratio, 0.46), and OS (18.2 vs. 8.3 months; hazard ratio, 0.48). These results align with those of the overall HER2-low patient population (including patients with HR-positive and HR-negative disease) in the DESTINY-Breast04 trial (Table 1) [6].

### 2.4. Evidence for T-DXd in HER2-Ultralow Breast Cancer

T-DXd has shown promising activity in 2 clinical trials, which included a subgroup of patients with HER2-ultralow metastatic breast cancer. In the phase 2 DAISY trial (NCT04132960), 15 patients from cohort 3 were determined to have tumors with HER2-ultralow expression levels, and a confirmed ORR of 40% (6/15 patients) was observed [7]. The DESTINY-Breast06 trial included 152 patients with HR-positive, HER2-ultralow metastatic breast cancer (T-DXd, *n* = 76; TPC, *n* = 76), and an exploratory subgroup analysis was conducted in this population [8]. T-DXd demonstrated efficacy with a clinically meaningful improvement in the HER2-ultralow population compared with TPC, similar to the efficacy observed in the HER2-low population (Table 1). Median PFS by BICR was 13.2 months in the T-DXd arm compared with 8.3 months in the TPC arm (hazard ratio, 0.78; 95% CI, 0.50–1.21); ORR was 61.8% versus 26.3%, respectively [8]. Given that this represented an exploratory subgroup analysis, efficacy was not statistically tested [8].

## 3. How Should T-DXd Treatment Be Sequenced for HER2-Low and HER2-Ultralow Advanced/Metastatic Breast Cancer?

Based on the results from DESTINY-Breast04, T-DXd (5.4 mg/kg) is approved for the treatment of adult patients with unresectable or metastatic HER2-low breast cancer (regardless of HR status), as determined by a US Food and Drug Administration (FDA) approved test, who have received prior chemotherapy in the metastatic setting or developed disease recurrence during or within 6 months of completing adjuvant chemotherapy [57]. Due to the results from the DESTINY-Breast06 trial, T-DXd has been approved for the treatment of patients with HR-positive, HER2-low or HER2-ultralow advanced breast cancer, as determined by a locally or regionally approved test, that has progressed on one or more endocrine therapies in the metastatic setting [10].

The National Comprehensive Cancer Network (NCCN) Clinical Practice Guidelines in Oncology (NCCN Guidelines^®^) for Breast Cancer recommend either systemic chemotherapy (category 1, preferred regimen) or T-DXd (other recommended regimen) as a first-line treatment option for HR-positive, HER2-low or HER2-ultralow breast cancer with visceral crisis or endocrine-refractory breast cancer [29]. Since the response rates with T-DXd were higher compared with single-agent chemotherapy, oncologists may steer toward T-DXd when more rapid symptomatic control is needed. T-DXd is also recommended as an “other recommended regimen” in the second-line setting for HR-negative, HER2 IHC 1+, or 2+/ISH− metastatic breast cancer with no germline *BRCA1*/*2* mutation [29].

### 3.1. What Other ADCs Can Be Considered for HER2-Negative Breast Cancer (Inclusive of HER2-Low and HER2-Ultralow)?

Patients with HER2-low and HER2-ultralow breast cancer are eligible to receive other ADCs that have been approved for HER2-negative breast cancer [27,28,31]. Sacituzumab govitecan (SG) is a trophoblast cell surface antigen 2 (TROP2)–directed ADC that is approved for the treatment of patients with unresectable locally advanced or metastatic HR-positive, HER2-negative breast cancer who have received ET and ≥2 additional systemic therapies in the metastatic setting [58,59]. The payload of SG is an active metabolite of irinotecan called SN38, which is a derivative of camptothecin. Like DXd, SN38 is a topoisomerase I inhibitor [51,52,53].

SG received approval for the treatment of advanced HR-positive, HER2-negative breast cancer based on findings from the TROPiCS-02 study (NCT03901339). At DCO for the primary analysis (3 January 2022), the median PFS by BICR was 5.5 months for SG and 4.0 months for TPC (eribulin, vinorelbine, capecitabine, or gemcitabine; hazard ratio, 0.66; *p* = 0.0003) [59]. At DCO for the final analysis (1 July 2022), the median OS for the SG cohort was 14.4 months versus 11.2 months for TPC (hazard ratio, 0.79 [95% CI, 0.65–0.96]; *p* = 0.020), and the ORR was 21% for SG versus 14% for TPC [60].

SG is also approved for the treatment of patients with metastatic TNBC after ≥2 lines of chemotherapy, at least one of which was for the treatment of metastatic disease [58]. In the phase 3 ASCENT trial (NCT02574455), patients with relapsed or refractory metastatic TNBC received either SG or TPC (eribulin, vinorelbine, capecitabine, or gemcitabine); at DCO (11 March 2020), the median PFS by BICR was 5.6 months with SG versus 1.7 months with TPC (hazard ratio, 0.41; [95% CI, 0.32–0.52]; *p* < 0.001), the median OS was 12.1 months with SG and 6.7 months with TPC (hazard ratio, 0.48 [95% CI, 0.38–0.59]; *p* < 0.001), and the ORR was 35% and 5% for SG and TPC, respectively [61].

The NCCN Guidelines^®^ for Breast Cancer recommend SG as a category 1 preferred second-line treatment option for HR-positive, HER2-negative metastatic breast cancer if the patient is not eligible for T-DXd. SG may be used after prior endocrine therapy, a CDK4/6i, and at least two lines of chemotherapy, one of which was a taxane, and at least one of which was in the metastatic setting. SG is also a category 1 preferred second-line treatment option for any HR-negative, HER2-negative breast cancer [29].

Another TROP2-directed ADC, datopotamab deruxtecan (Dato-DXd), is approved for the treatment of patients with HR-positive, HER2-negative breast cancer who have received prior endocrine-based therapy and chemotherapy for unresectable or metastatic disease [62]. Approval was based on the global, randomized, phase 3 TROPION-Breast01 study (NCT05104866), which reported an improved PFS by BICR with Dato-DXd compared with chemotherapy (6.9 and 4.9 months, respectively; hazard ratio, 0.63; *p* < 0.0001) [63]. However, median OS did not differ significantly between treatment groups (18.6 and 18.3 months, respectively; hazard ratio, 1.01; *p* = 0.94) [64]. The NCCN Guidelines^®^ for Breast Cancer recommend Dato-DXd as a second-line treatment option after chemotherapy or a PARPi for patients with HR-positive, HER2-negative disease if the patient is not a candidate for T-DXd [29].

### 3.2. How Should ADCs Be Sequenced in HER2-Negative Breast Cancer (Inclusive of HER2-Low and HER2-Ultralow)?

Retrospective studies exploring the effectiveness of sequential ADCs in individuals with HER2-negative metastatic breast cancer have demonstrated that treatment with a second ADC, regardless of the specific treatment, results in reduced efficacy, including substantially shorter PFS [65,66,67,68,69]. This may be due to cross-resistance that can develop against the antibody or payload component of ADCs [66,70]. The approved DXd ADCs and SG have payloads with a similar mechanism of action (topoisomerase inhibition), which raises questions about the sequencing of these therapies. A multi-institutional study of patients receiving multiple ADCs for metastatic breast cancer observed that cross-resistance to the second ADC could be driven by either the antibody target or the payload [71]. A subset of patients was identified who had variants in topoisomerase I–associated genes (*TOP1*, *TOP2A*, *TOP3A*, *TOP3B*), which could mediate cross-resistance to a second ADC with a topoisomerase I inhibitor payload.

In the absence of robust clinical evidence, the optimal sequencing for ADCs remains uncertain, and extensive research is required to determine sequencing strategies and identify resistance mechanisms to ADCs [5,72,73]. The prospective TRADE-DXd study (NCT06533826) aims to address this need. In this study, patients with HER2-low metastatic breast cancer who have received no or one previous line of chemotherapy will be treated with T-DXd or Dato-DXd and switched to the opposite ADC upon disease progression to improve knowledge of optimal treatment sequencing and mechanisms of resistance [73].

## 4. What Is the Efficacy of T-DXd in Patients with HER2-Low Metastatic Breast Cancer with Brain Metastases?

Brain metastases (BMs) may develop in approximately 8.5–15.0% of patients with HER2-low metastatic breast cancer [74,75]. The systemic and intracranial efficacy of T-DXd in patients with HER2-low metastatic breast cancer and BMs has been investigated in subgroup analyses and a single-arm phase 2 study [76,77,78]. In a subgroup analysis of the DESTINY-Breast04 trial, patients with HER2-low metastatic breast cancer with investigator-assessed BMs at baseline had a longer median PFS when treated with T-DXd (8.1 months; *n* = 24) compared with TPC (4.8 months; *n* = 8; hazard ratio, 0.71) [76]. Another DESTINY-Breast04 subgroup analysis in patients with HER2-low metastatic breast cancer also demonstrated the intracranial efficacy of T-DXd. Patients treated with T-DXd (*n* = 24) had a greater intracranial ORR (25.0% vs. 0%), clinical benefit rate (58.3% vs. 18.2%), and disease control rate (75.0% vs. 63.6%) versus those given TPC (*n* = 11) [77]. In the prospective, single-arm, phase 2 DEBBRAH trial (NCT04420598), the efficacy and safety of T-DXd was assessed in patients with HER2-low advanced breast cancer with asymptomatic untreated or progressing BMs after local therapy (*n* = 12). Overall, the intracranial ORR and intracranial clinical benefit rate per Response Assessment in Neuro-Oncology Brain Metastases (RANO-BM) was 41.7% and 58.3%, respectively. The median intracranial time to response was 2.3 months, median intracranial duration of response (DOR) was 7.2 months, and median PFS was 5.4 months [78].

## 5. Testing: How Do I Identify Patients Who Are Eligible for Treatment?

### 5.1. Which Test(s) Should I Use to Determine if a Patient Has HER2-Low or HER2-Ultralow Breast Cancer?

The NCCN Guidelines^®^ for Breast Cancer and ASCO–CAP clinical practice guidelines recommend that all patients with new primary or newly metastatic breast cancer undergo tumor HER2 expression testing to guide therapy choices [2,3,29]. HER2 testing by IHC is the primary method to identify patients who may benefit from HER2-directed agents [2,3,29]. IHC has several advantages as a diagnostic tool, including rapid turnaround time, wide availability, and the ability to assess intratumoral heterogeneity [9,79,80]. *HER2* (*ERBB2*) gene amplification, which is associated with HER2 overexpression, is assessed by ISH [81,82]. ISH is used as a reflex test for tumors with HER2 equivocal status (IHC score of 2+) that classifies them as HER2-positive (IHC 2+/ISH+) or, per the suggested HER2 spectrum classification, HER2-low (IHC 2+/ISH−) [3,5].

The DESTINY-Breast04 and DESTINY-Breast06 trials used the VENTANA anti-HER2/neu (4B5) rabbit monoclonal primary antibody (Roche), which has been clinically validated and is currently the only FDA-approved companion diagnostic assay for the identification of patients with HER2-low and HER2-ultralow metastatic breast cancer who may be eligible for T-DXd treatment [6,8,83,84].

While VENTANA HER2 4B5 is the most commonly used HER2 IHC assay in the United States, pathologists may consider non-4B5 assays after weighing the available evidence on the reliability of identifying patients for T-DXd treatment [5,25,85]. The standardization of protocols and both positive and negative controls are also important factors for pathologists to consider [2,3,86]. The awareness of the influences of preanalytical variables (e.g., sample fixation, temperature, reaction time, antigen retrieval, substrate concentration) and heterogeneous HER2 expression are critical [5,85,87,88].

In addition to selecting the optimal assay, scoring details within pathology reports and clinical notes will be important to facilitate the identification of potential candidates for therapy. The updated CAP Breast Biomarker Reporting Template (version 1.6.0.0) now includes definitions of HER2-low (IHC score 1+ or 2+/ISH−) and HER2-ultralow (defined by the CAP Breast Biomarker Reporting Template as an IHC score of 0 with membrane staining that is incomplete and that is faint/barely perceptible in less than or equal to 10% of tumor cells [IHC 0+/with membrane staining]) [9]. Best practices for pathologists include reporting discrete IHC scores to guide eligibility for T-DXd, while also being mindful that the reported IHC result potentially impacts treatment-related survival outcomes for patients [5].

Alternative complementary methods to assess HER2 expression and identify HER2-low and HER2-ultralow breast cancer, including quantitative mRNA and protein-based assays and the use of artificial intelligence to interpret the results, are being explored [89,90,91,92,93]. Until clear parameters are established for the use of other approaches to classify HER2-low and HER2-ultralow breast cancer, IHC with reflex-ISH, as needed, remains the gold standard [13,87].

### 5.2. What Are Some of the Challenges of Testing and Identifying HER2-Low and HER2-Ultralow Breast Cancer?

HER2 IHC assays were initially developed and validated to identify patients with HER2-positive breast cancer [82,94]. However, with the FDA approval of T-DXd for HER2-low and HER2-ultralow metastatic breast cancer, careful evaluation of low HER2 expression levels has become clinically relevant [2,95]. Specifically, there is now a critical need to distinguish present (IHC 1+, IHC 0 with membrane staining) and absent (IHC 0 absent) HER2 surface membrane expression [5,9]. The 2023 update to the ASCO–CAP guideline for HER2 testing in breast cancer recommends that testing laboratories distinguish IHC 0 from IHC 1+ and suggests several best practices to do so (Table A1) [2].

Accurate and consistent testing and reporting methods are important to ensure that HER2 expression levels in breast cancer are reliably detected and classified [13,85,96]. Variability in the sensitivity and specificity of commercially available HER2 IHC assays has been reported and can lead to discordant results [86,97,98]. Concordance between the VENTANA HER2 4B5 assay and non-4B5 assays tends to be higher with HER2 IHC 3+ samples and lower when used to identify samples as HER2-low or HER2 IHC 0 [99,100]. A study assessing agreement between the VENTANA HER2 4B5 assay and other assays when classifying samples as HER2-low versus HER2 IHC 0 found positive and negative percentage agreements of 87.5% and 61.9%, respectively [100].

Additionally, even in closely controlled cases with standardized assays, scoring and interpretation rely on visual assessment, which may introduce additional variability [22,86]. HER2 classification of the same sample can differ between laboratories and individual pathologists [16,88,100]. A study of 400 patient samples previously categorized as HER2 IHC 0 reported 28.3% being rescored as HER2-ultralow and 37.5% being rescored as IHC 1+ by the same pathologists after a 2–10 week washout period [101]. In another study, concordance of HER2 expression classification among pathologists, especially in the IHC 0 and 1+ range, was reported to be as low as 26% [88]. A caveat in both the above cited studies was that the pathologists did not receive any training in scoring samples with ultralow IHC levels [88,101]. Pathologists can benefit from a thorough understanding of the clinical impact that HER2 status designation has on treatment decisions and ultimately patient outcomes [85,87,88].

### 5.3. How Variable Is HER2 Expression in Breast Cancer?

Repeat tumor testing to account for HER2 heterogeneity or changes in HER2 expression upon disease progression may be justified to inform therapeutic decision-making [102,103,104]. ASCO–CAP guidelines recommend tumor testing for HER2 in all patients with newly diagnosed breast cancer, and additionally, at the development of metastatic disease [3]. Beyond this, intratumoral variability in HER2 expression could lead to inconsistent IHC results, which have been reported in up to 40% of breast cancers, and is more common in HER2-low and HER2-equivocal (IHC 2+) cases than in HER2 IHC 3+ [102,105]. Classification of individual samples taken from breast tumors and metastases during postmortem biopsies demonstrated that HER2-low and HER2 IHC 0 lesions coexisted at end-stage disease in 80% of patients [106]. HER2 expression can also change upon disease relapse or progression [23,107,108,109]. When comparing classification between biopsy samples collected from primary tumors to those collected from metastases, changes from HER2 IHC 0 to HER2-low and vice versa have been reported in up to 50% of cases [107,110,111,112,113]. These conversions occurred in both HR-positive and HR-negative tumors, but conversions were reported more commonly in HR-positive tumors [23,107,108]. In addition, biopsy samples obtained after relapse or from distant metastases have shown shifts toward HER2-low status compared with the original HER2 IHC 0 primary tumors [23,107,108].

### 5.4. How Should I Evaluate HER2 Status in Patients with a HER2 IHC 0 Tumor Score on Their Most Recent Biopsy?

In cases where tumors were previously scored as HER2 IHC 0, retesting a prior biopsy or acquiring a new biopsy may be performed to reevaluate HER2 status and identify patients with HER2-ultralow tumors who may benefit from targeted treatment [5,114]. While there are limited data on the value of multiple or repeat biopsies for identifying HER2-ultralow disease, some evidence suggests that repeat biopsies can reclassify HER2 IHC 0 breast cancer as HER2-low in some instances [104,115]. Further evaluation is needed to clearly establish the value of repeat biopsy in this scenario.

Upon request, pathologists may be able to re-review HER2-stained slides or re-stain archived tissue if available. These methods may circumvent the need for a new biopsy. Communication between oncologists and pathologists is essential when evaluating for HER2-ultralow status. Multidisciplinary tumor boards can be ideal for detailed discussions and reviews.

## 6. Safety: What Are the Best Practices for Preventing, Monitoring, and Managing Adverse Events with T-DXd?

In the phase 3 DESTINY-Breast04 (N = 371) and DESTINY-Breast06 (N = 434) trials, the safety profile of T-DXd (5.4 mg/kg) was generally manageable in patients with HER2-low/HER2-ultralow advanced breast cancer [6,8,56]. Patient-reported outcomes indicated that global health status and patient quality of life were maintained with T-DXd [116,117,118].

The most common drug-related treatment-emergent adverse events (AEs) in patients with HER2-low and HER2-ultralow advanced breast cancer were low grade and generally gastrointestinal or hematologic in nature [6,8,56]. T-DXd–related interstitial lung disease (ILD)/pneumonitis, a potentially serious and life-threatening AE, was observed in some patients. ILD occurrences were mostly low grade; however, fatalities have occurred [6,8,56]. It is crucial to monitor for and manage this condition to minimize serious or even fatal outcomes [119,120]. Here, we will focus on ILD/pneumonitis, nausea/vomiting, fatigue, and alopecia, as these AEs are the most common and/or pose a significant impact to patients’ quality of life.

### 6.1. What Are the Best Practices for Preventing, Monitoring, and Managing ILD/Pneumonitis with T-DXd?

Most ILD/pneumonitis events with T-DXd are of low grade (grade ≤ 2), although fatal events have occurred [6,8,119,121]. In the primary analysis of DESTINY-Breast04, any-grade adjudicated T-DXd–related ILD/pneumonitis occurred in 45 patients (12.1%), with 13 (3.5%) grade 1, 24 (6.5%) grade 2, 5 (1.3%) grade 3, and 3 (0.8%) grade 5 events [6]. In DESTINY-Breast06, any-grade adjudicated T-DXd–related ILD/pneumonitis was reported in 49 patients (11.3%); 7 (1.6%) were grade 1, 36 (8.3%) were grade 2, 3 (0.7%) were grade 3, and 3 (0.7%) were grade 5 [8]. ILD/pneumonitis can develop at any point during treatment; however, a pooled analysis of 9 phase 1 and 2 T-DXd (≥5.4 mg/kg) monotherapy studies showed that the median time to adjudicated drug-related ILD/pneumonitis onset was 5.4 months (range, <0.1–46.8 months), and 87% of cases occurred during the first year of T-DXd treatment [122]. These results are similar to previous reports with T-DXd therapy [6,8,123].

**Figure 2:**
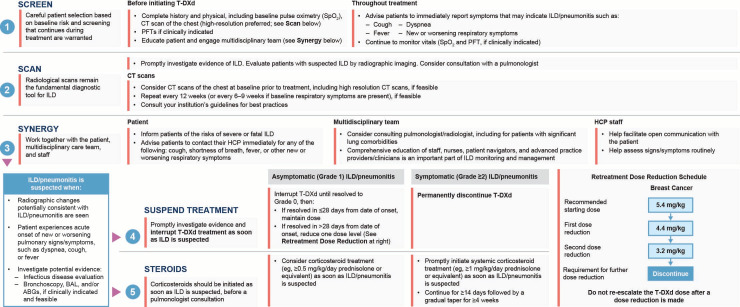
Early identification of ILD/pneumonitis using the 5 “S” strategy [121,128,129]. ABG, arterial blood gas; BAL, bronchoalveolar lavage; CT, computed tomography; HCP, health care professional; ILD, interstitial lung disease; PFT, pulmonary function test; SpO_2_, peripheral capillary oxygen saturation; T-DXd, trastuzumab deruxtecan.

Before receiving T-DXd, patients must be thoroughly evaluated to identify individual risk factors for ILD/pneumonitis (Figure 2) [124]. Risk factors for T-DXd–related ILD/pneumonitis have yet to be fully established and remain under investigation [121,122,125,126]. Clinical factors of interest include the presence of concurrent lung comorbidities, moderate/severe renal impairment (moderate, serum creatine clearance >30 to <60 mL/min; severe, <30 mL/min), time since initial diagnosis (>4 years), baseline oxygen saturation (SpO_2_; <95%), >5.4 mg/kg T-DXd dose, and age >65 years [5,121,122,125]. Future research should be aimed at characterizing biomarkers to help identify patients who might be at higher risk of developing ILD/pneumonitis [127].

Patient, caregiver, and health care provider education is critical to ensure prompt symptom recognition, diagnosis, and appropriate management of ILD/pneumonitis events to minimize serious outcomes [121]. Grade 1 ILD/pneumonitis is asymptomatic and identified on a CT scan, while grade ≥ 2 events are symptomatic [124,125]. ILD/pneumonitis can present with radiographic opacities [121]. Proactive surveillance is important to identify cases of ILD/pneumonitis, interrupt therapy, and implement prompt interventions. This proactive monitoring should include CT scans of the chest at intervals of 6–12 weeks during treatment, and patients should be advised to immediately report any symptoms suggestive of ILD/pneumonitis, such as dyspnea, cough, or fever [5,121,128]. Involvement of the entire multidisciplinary team, including nurses, advanced practice providers, pulmonologists, and radiologists, in monitoring for signs and symptoms of ILD/pneumonitis may help to ensure that this potential AE is diagnosed and addressed as early as possible (Figure 2) [121,128].

Guidelines for the monitoring and management of ILD/pneumonitis, which have been discussed in detail in other publications [119,121,128,129], emphasize that T-DXd treatment should be interrupted if ILD/pneumonitis is suspected. Further evaluations should be performed to confirm or rule out an ILD/pneumonitis diagnosis if the patient develops radiographic changes consistent with ILD/pneumonitis or develops an acute onset of new or worsening pulmonary or other related signs/symptoms [119,121,129]. Corticosteroids should be initiated as soon as grade 1 ILD is suspected, and a pulmonary consultation should be considered. For grade 1 ILD, patients should be started on systemic corticosteroids such as ≥0.5 mg/kg/day prednisolone or equivalent until there is improvement followed by a gradual taper over ≥4 weeks. For grade 2 ILD, systemic corticosteroids such as ≥1 mg/kg/day prednisolone or equivalent should be initiated for ≥14 days or until complete resolution, followed by gradual taper over ≥4 weeks. For grade ≥3 ILD, higher doses of empiric corticosteroids may be considered, such as methylprednisolone IV treatments (500–1000 mg/day for 3 days; Figure 2) [119,121,129].

A pooled analysis of 9 T-DXd clinical trials in 1150 patients with breast, gastric, colorectal, or non–small cell lung cancer suggested that patients with grade 1 ILD may be retreated with T-DXd upon complete radiographic recovery [122]. Of the 47 patients who were retreated with T-DXd, 3 patients had a recurrence of ILD/pneumonitis [122]. A retrospective multi-institutional study reported that among 38 patients with grade 1 ILD who were rechallenged, 10 (26%) developed recurrent ILD (seven grade 1, two grade 2, and one grade 3 event). Of the 9 patients with grade 2 ILD who were rechallenged with T-DXd, 2 (22%) developed recurrent ILD (one grade 1, and one grade 3 event) [130]. Further research is needed to understand the risks and benefits of T-DXd retreatment in patients who have asymptomatic ILD/pneumonitis that has not resolved or patients with grade 2 ILD/pneumonitis that has fully resolved.

### 6.2. What Are the Best Practices for Preventing and Managing Nausea/Vomiting with T-DXd?

The NCCN Guidelines^®^ for Antiemesis categorize T-DXd as being of high emetic risk [57,131]. In the DESTINY-Breast04 trial, any-grade drug-related nausea and vomiting were reported in 73.0% and 34.0%, respectively [6]. In the DESTINY-Breast06 trial, 65.9% of patients experienced any-grade drug-related nausea, while 27.2% experienced vomiting with T-DXd [8].

**Figure 3:**
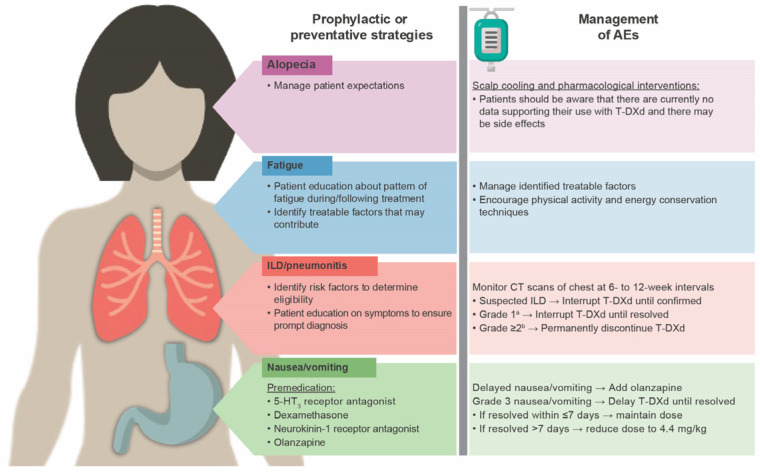
Monitoring and management of T-DXd–related AEs [5,119,121,124,132]. 5-HT_3_, serotonin receptor; AE, adverse event; CT, computed tomography; ILD, interstitial lung disease; T-DXd, trastuzumab deruxtecan. ^a^ Asymptomatic, radiographic findings only. ^b^ Grade 2: Symptomatic, mild respiratory symptoms that do not interfere with daily living. Grade 3: Symptomatic, interfering with daily living, possibly needing oxygen therapy. Grade 4: Severe, disabling symptoms leading to hospitalization and ventilatory support. Grade 5: Death.

Premedication with a regimen that includes a 5-HT_3_ receptor antagonist such as ondansetron or palonosetron, dexamethasone, a neurokinin-1 receptor antagonist, and olanzapine is advised to prevent nausea and vomiting; olanzapine can also be administered for delayed nausea and vomiting (Figure 3) [5,119,124,132]. In the recently published ERICA study, addition of oral olanzapine 5 mg/day for 6 days to a 5-HT_3_ receptor antagonist and dexamethasone appeared to be effective in preventing delayed (24–120 h) and persistent (120–504 h) nausea and vomiting in patients receiving their first cycle of T-DXd treatment [133]. However, a recent survey indicated that these medications are underused, noting that 37% of patients treated with ADCs did not receive an antiemetic prophylactic regimen [134]. According to clinical trial protocol management guidelines, T-DXd dosing should be delayed for cases of grade 3 nausea until resolved to grade ≤ 1; if resolved in ≤7 days from day of onset, T-DXd dose can be maintained; if resolved in >7 days from day of onset with optimal antiemetic use, T-DXd dose should be reduced by 1 dose level [6,119].

### 6.3. What Are the Best Practices for Preventing, Monitoring, and Managing Fatigue with T-DXd?

In the DESTINY-Breast04 trial, 47.7% of patients in the T-DXd arm experienced fatigue [6]; the corresponding percentage was 46.8% in the DESTINY-Breast06 trial [8]. Patients should undergo a thorough assessment using validated tools to identify treatable factors that may contribute to fatigue and follow appropriate treatment guidelines to manage any identified factors. Educating and counseling patients and their families/caregivers about the known pattern of fatigue during and following treatment is beneficial. Implementing supportive measures such as starting or maintaining physical activity, optimizing diet, prioritizing mental health, and other energy conservation techniques can also be useful (Figure 3) [119,124,135].

### 6.4. What Are the Best Practices for Preventing, Monitoring, and Managing Alopecia with T-DXd?

In DESTINY-Breast04 and DESTINY-Breast06, alopecia occurred in 37.7% and 45.4% of patients, respectively [6,8]. T-DXd–related alopecia tends to be grade 1 (<50% hair loss not requiring a wig/hair piece to camouflage) [119]. There is ongoing research in the use of scalp cooling for chemotherapy-induced alopecia (hair loss) prevention [136]; however, there are currently no data supporting the use of scalp cooling and pharmacologic interventions to prevent T-DXd–related alopecia. Patients should be advised that these interventions are still under investigation as they may come with burdensome logistics (such as availability of devices), cost implications (inconsistent insurance coverage), and potential side effects (Figure 3) [119,124,137].

### 6.5. How Safe Is T-DXd in Older Patients?

Older patients undergoing chemotherapy are more likely than younger patients to experience AEs due to age-related physiological changes and altered pharmacokinetics of chemotherapeutic agents [138]. Despite exhibiting similar disease characteristics and regardless of treatment, women aged ≥70 years with HER2-positive advanced breast cancer experienced increased rates of AEs compared with those aged <70 years [139]. Of 1287 patients with HER2-positive or HER2-low breast cancer enrolled in T-DXd clinical trials, 22% were ≥65 years of age. No overall difference in efficacy was observed between patients aged ≥65 years compared with younger patients; however, a higher incidence of grade 3 or 4 AEs was observed in patients aged ≥65 years (59% vs. 49%, respectively) [57,140]. Additional studies on the use of T-DXd and how to mitigate toxicity in patients of different ages with HER2-low and HER2-ultralow breast cancer may be beneficial.

## 7. Evolving Treatment Paradigm

### 7.1. HR-Positive, HER2-Low Breast Cancer

T-DXd should be considered following ET for patients with HR-positive, HER2-low breast cancer, particularly those with rapid progression on or after ET or visceral crisis, given the demonstrated PFS, ORR, and DOR advantage of T-DXd over TPC in DESTINY-Breast06 [8]. Mature OS data to confirm the benefit of T-DXd in this population are pending. When determining treatment sequence following ET strategies, it is vital to balance efficacy with risk, potential toxicity (including high-grade ILD/pneumonitis), patient goals, and quality of life.

### 7.2. HER2-Ultralow Breast Cancer

Data from exploratory analyses in DESTINY-Breast06, which included patients with HR-positive, HER2-ultralow breast cancer, are promising and emphasize the need to further standardize HER2-ultralow definitions and testing practices. Standardization is crucial to ensure appropriate patient access to therapy and to prevent disparities in cancer care due to inconsistent pathology results. Education on HER2 classification will be important as HER2-ultralow becomes another clinically relevant breast cancer subtype.

## 8. Future Directions

Ongoing and future clinical trials will continue to explore the efficacy and safety of T-DXd monotherapy and combination regimens in earlier treatment lines for patients with HER2-low or HER2-ultralow metastatic breast cancer (Table 2). Additionally, the possibility that some patients whose tumors express HER2 levels below the current limits of detection (e.g., HER2 IHC 0 absent membrane staining) might benefit from T-DXd treatment is also under investigation [141]. Future studies will also address the real-world efficacy, safety, treatment patterns, and cost-effectiveness of T-DXd in HER2-low and HER2-ultralow breast cancer. Finally, further research aims to improve ILD/pneumonitis assessment and confirmation, including monitoring for recurrence if therapy is rechallenged after recovery.

The efficacy and safety of several other HER2- and TROP2-directed ADCs are also currently being assessed for metastatic breast cancers expressing low levels of detectable HER2 by IHC. Other HER2-directed ADCs in early-phase clinical trials include trastuzumab duocarmazine, disitamab vedotin, DHES0815A, BB-1701, DB-1303, and ARX788 [142,143,144,145,146,147]. These HER2-directed ADCs have payloads with different mechanisms of action compared with T-DXd, potentially providing additional options for treatment sequencing.

Regarding TROP2-directed ADCs, Dato-DXd has been approved in patients with HR-positive, HER2-negative (IHC 0, IHC 1+, IHC2+/ISH−) breast cancer who had previously had ET and chemotherapy [62,63,64]. Dato-DXd is also being evaluated compared with chemotherapy in patients with TNBC who are not candidates for PD-L1 inhibitor therapy in the TROPION-Breast02 trial (NCT05374512) [148]. A high response rate was reported with the combination of Dato-DXd and durvalumab in the first-line treatment setting in the phase 1b/2 BEGONIA trial (NCT03742102) [149]. This provided the rationale for additional ongoing studies assessing the combination in patients with TNBC who have received neoadjuvant therapy (TROPION-Breast03; NCT05629585), who are treatment-naive (TROPION-Breast04; NCT06112379), or in patients with locally recurrent, inoperable or metastatic, PD-L1 positive TNBC not previously treated with chemotherapy or targeted systemic anticancer therapy (TROPION-Breast05; NCT06103864) [150,151,152].

Studies investigating the efficacy of SG in earlier-line settings and chemotherapy-naive patients are also currently underway [49,153]. In the ASCENT-04/KEYNOTE-D19 trial, SG combined with pembrolizumab showed a significant improvement in PFS by BICR compared with chemotherapy and pembrolizumab (hazard ratio, 0.65; 95% CI, 0.51–0.84; *p* = 0.0009) in patients with previously untreated, PD-L1–positive locally advanced unresectable or metastatic TNBC [49].

Finally, additional research is needed on the sequence of ADC treatments for patients with HER2-low metastatic breast cancer, an area that will be addressed in part by the TRADE-DXd study (Table 2).

**Table 2:** Summary of selected clinical trials investigating T-DXd in HER2-low, HER2-ultralow, or HER2 IHC 0 absent membrane staining breast cancer.

Trial, Treatment	Study Phase	NCT	Patient Cohort	Efficacy EndPoint(s)
DAISY, T-DXd	2	NCT04132960	HER2-positive, HER2-low, or HER2 IHC 0 metastatic breast cancer. Patients must have received ≥1 line of chemotherapy in the metastatic setting. Prior treatment should have included anthracyclines and/or taxanes.	Primary endpoint: confirmed ORR [7]
DEBBRAH, T-DXd	2	NCT04420598	HER2-low metastatic breast cancer with CNS involvement (BM progression after local treatment or LMC).	Primary endpoints: intracranial ORR [154]
DESTINY-Breast08, T-DXd + combinations	1b	NCT04556773	HR-positive, HER2-low metastatic breast cancer	Secondary endpoints: ORR, PFS, DOR, OS [155]
DESTINY-Breast15, T-DXd	3b	NCT05950945	HR-positive or HR-negative, HER2-low or HER2 IHC 0 metastatic breast cancer. Patients must have received 1 or 2 prior lines of therapy.	Primary endpoint: TTNT [141,156]
DESTINY-Breast Respond HER2-low Europe	Observational, prospective	NCT05945732	HER2-low unresectable and/or metastatic breast cancer. Patients must have received at least 1 prior chemotherapy in the metastatic setting or experienced disease recurrence within 6 months after adjuvant chemotherapy.	Primary endpoint: rwTTNT [157]
PONTIAC	2	NCT06486883	HR-positive HER2-low or HER2-ultralow unresectable and/or metastatic breast cancer. Patients must not have received prior treatment with any systemic therapy for advanced disease.	Primary endpoint: PFS [158]
TUXEDO-4, T-DXd	2	NCT06048718	HER2-low breast cancer with active BMs. Patients must have received ≥1 line of systemic therapy in the advanced setting.	Primary endpoint: ORR [159,160]
TRADE-DXd, Treatment sequences of T-DXd and Dato-DXd	2	NCT06533826	Patients with HR-positive or HR-negative, HER2-low tumors. Patients must have received 0–2 lines of chemotherapy, depending on patient cohort^a^	Primary endpoint: ORR Secondary endpoints: PFS, OS [161]

BM, brain metastasis; CNS, central nervous system; Dato-DXd, datopotamab deruxtecan; DOR, duration of response; HER2, human epidermal growth factor receptor 2; HR, hormone receptor; IHC, immunohistochemistry; LMC, leptomeningeal carcinomatosis; ORR, objective response rate; OS, overall survival; PFS, progression-free survival; rwTTNT, real-world time to next treatment; T-DXd, trastuzumab deruxtecan; TTNT, time to next treatment. ^a^ ADC1 T-DXd and Dato-DXd cohorts: 0–1 prior lines of chemotherapy in the metastatic setting; ADC2 T-DXd cohort: 1–2 prior lines of chemotherapy including Dato-DXd as the most recent therapy; ADC2-Dato-DXd cohort: 1–2 prior lines of chemotherapy including T-DXd as the most recent therapy.

## 9. Conclusions

With the approval of T-DXd for HER2-low and HER2-ultralow metastatic breast cancer, careful evaluation of low HER2 expression levels has become clinically relevant. Consensus on HER2-ultralow definitions and HER2 testing standardization is essential for the timely identification and treatment of patients with HER2-low or HER2-ultralow breast cancers who may benefit from T-DXd therapy.

Clinicians will need to weigh efficacy with patient goals, quality of life, and potential toxicity to make the best risk-benefit and treatment sequencing decisions for each patient. Ongoing and future clinical trials will further inform clinical decision-making for patients with advanced or metastatic HER2-low and HER2-ultralow breast cancer and provide information on optimal sequencing strategies for HER2- and TROP2-directed ADCs in these populations.

## Figures and Tables

**Table 1:** Efficacy outcomes with T-DXd in DESTINY-Breast04 and DESTINY-Breast06.

DESTINY-Breast04 ^a,b^		HR-Positive Cohort *n* = 494		HR-Negative Cohort *n* = 58		All Patients *n* = 557	
	Efficacy Endpoint	T-DXd	TPC	T-DXd	TPC	T-DXd	TPC
**Primary analysis **[6] **DCO, 11 January 2022**	**N**	**331**	**163**	**40**	**18**	**373**	**184**
	ORR by BICR ^c^ (95% CI), %	52.6 (47.0–58.0)	16.3 (11.0–22.8)	50.0 (33.8–66.2)	16.7 (3.6–41.4)	52.3 (47.1–57.4)	16.3 (11.3–22.5)
	PFS by BICR, median (95% CI), months	10.1 (9.5–11.5)	5.4 (4.4–7.1)	8.5 (4.3–11.7)	2.9 (1.4–5.1)	9.9 (9.0–11.3)	5.1 (4.2–6.8)
	Hazard ratio (95% CI) *p* value	0.51 (0.40–0.64) *p* < 0.001		0.46 (0.24–0.89) ^c^		0.50 (0.40–0.63) *p* < 0.001	
	OS, median (95% CI), months	23.9 (20.8–24.8)	17.5 (15.2–22.4)	18.2 (13.6–NE)	8.3 (5.6–20.6)	23.4 (20.0–24.8)	16.8 (14.5–20.0)
	Hazard ratio (95% CI) *p* value	0.64 (0.48–0.86) *p* = 0.003		0.48 (0.24–0.95) ^d^		0.64 (0.49–0.84) *p* = 0.001	
**DESTINY-Breast06**		**HER2-Low Population ** ***n *** **= 713**		**HER2-Ultralow Population ** ***n *** **= 152**		**ITT Population ^e^** ***n *** **= 866**	
	**Efficacy Endpoint**	**T-DXd**	**TPC**	**T-DXd**	**TPC**	**T-DXd**	**TPC**
**Primary analysis **[8] **DCO, 18 March 2024**	**N**	**359**	**354**	**76**	**76**	**436**	**430**
	ORR by BICR, (95% CI), %	56.5 (51.2–61.7)	32.2 (27.4–37.3)	61.8 (50.0–72.8)	26.3 (16.9–37.7)	57.3 (52.5–62.0)	31.2 (26.8–35.8)
	PFS by BICR, median (95% CI), months	13.2 (11.4–15.2)	8.1 (7.0–9.0)	13.2 (9.8–17.3)	8.3 (5.8–15.2)	13.2 (12.0–15.2)	8.1 (7.0–9.0)
	Hazard ratio (95% CI) *p* value	0.62 (0.52–0.75) *p* < 0.001		0.78 (0.50–1.21)^d^		0.64 (0.54–0.76) *p* < 0.001	

BICR, blinded independent central review; DCO, data cutoff; HER2, human epidermal growth factor receptor 2; HR, hormone receptor; ITT, intention-to-treat; PFS, progression-free survival; ORR, objective response rate; OS, overall survival; T-DXd, trastuzumab deruxtecan; TPC, treatment of physician’s choice. ^a^ HR status based on data collected via the interactive web- and voice-response system at the time of randomization, which includes patients who were mis-stratified. ^b^ PFS and OS results reported in the long-term survival analysis of DESTINY-Breast04 were consistent with those reported in the primary analysis [6,56]. ^c^ Number of patients evaluated in the HR-positive HER2-low cohort for ORR was 333 for T-DXd and 166 for TPC; for this endpoint, HR status is based on data from the electronic data capture that was corrected for mis-stratification. ^d^ ITT population includes both HER2-low and HER2-ultralow populations. ^e^ *p* value was not evaluated in this exploratory analysis.

## Data Availability

Not applicable.

## Abbreviations

The following abbreviations are used in this manuscript:

5-HT_3_	Serotonin receptor
ADC	Antibody–drug conjugate
AE	Adverse events
ASCO	American Society of Clinical Oncology
BICR	Blinded independent central review
BMs	Brain metastases
CAP	College of American Pathologists
CDK4/6i	Cyclin-dependent kinase 4/6 inhibitor
CT	Computed tomography
Dato-DXd	Datopotamab deruxtecan
DCO	Data cutoff
DOR	Duration of response
DXd	Deruxtecan
ER	Estrogen receptor
ET	Endocrine therapy
FDA	US Food and Drug Administration
HER2	Human epidermal growth factor receptor 2
HR	Hormone receptor
IHC	Immunohistochemistry
ILD	Interstitial lung disease
ISH	In situ hybridization
ITT	Intention-to-treat
NCCN^®^	National Comprehensive Cancer Network^®^
ORR	Objective response rate
OS	Overall survival
PARPi	Poly (ADP-ribose) polymerase inhibitors
PD-1	Programmed cell death protein 1
PD-L1	Programmed death ligand 1
PFS	Progression-free survival
PR	Progesterone receptor
RANO-BM	Response Assessment in Neuro-Oncology Brain Metastases
SG	Sacituzumab govitecan
SpO_2_	Oxygen saturation
T-DM1	Trastuzumab emtansine
T-DXd	Trastuzumab deruxtecan
TNBC	Triple-negative breast cancer
TPC	Treatment of physician’s choice
TROP2	Trophoblast cell surface antigen 2

## Appendix A

**Table A1:** Best practices to distinguish between IHC 0 and IHC 1+ per 2023 ASCO–CAP guidelines.

**IHC interpretation**	Use standardized ASCO–CAP guidelines scoring criteria to examine HER2 IHC-stained slides
**Magnification**	When discriminating IHC 0 from IHC 1+ staining, examine HER2 IHC at high-power magnification (40×)
**Additional evaluation**	Consider second pathologist review when results are close to the IHC 0 versus IHC 1+ interpretive threshold (>10% of cells with incomplete membrane staining that is faint/barely perceptible)
**Assay controls**	Ensure the assay has an appropriate limit of detection by using controls with a range of protein expression (including IHC 1+)
**Preanalytic variables**	It is essential to consider preanalytic conditions of breast cancer tissue samples from both primary and metastatic sites

ASCO-CAP, American Society of Clinical Oncology-College of American Pathologists; HER2, human epidermal growth factor receptor 2; IHC, immunohistochemistry.

## References

[1] Harbeck N., Penault-Llorca F., Cortes J., Gnant M., Houssami N., Poortmans P., Ruddy K., Tsang J., Cardoso F. (2019). Breast cancer. Nat. Rev. Dis. Primers.

[2] Wolff A.C., Somerfield M.R., Dowsett M., Hammond M.E.H., Hayes D.F., McShane L.M., Saphner T.J., Spears P.A., Allison K.H. (2023). Human epidermal growth factor receptor 2 testing in breast cancer. Arch. Pathol. Lab. Med..

[3] Wolff A.C., Hammond M.E.H., Allison K.H., Harvey B.E., Mangu P.B., Bartlett J.M.S., Bilous M., Ellis I.O., Fitzgibbons P., Hanna W. (2018). Human epidermal growth factor receptor 2 testing in breast cancer: American Society of Clinical Oncology/College of American Pathologists clinical practice guideline focused update. J. Clin. Oncol..

[4] Ariga S. (2023). History and Future of HER2-Targeted Therapy for Advanced Gastric Cancer. J. Clin. Med..

[5] Tarantino P., Viale G., Press M.F., Hu X., Penault-Llorca F., Bardia A., Batistatou A., Burstein H.J., Carey L.A., Cortes J. (2023). ESMO expert consensus statements (ECS) on the definition, diagnosis, and management of HER2-low breast cancer. Ann. Oncol..

[6] Modi S., Jacot W., Yamashita T., Sohn J., Vidal M., Tokunaga E., Tsurutani J., Ueno N.T., Prat A., Chae Y.S. (2022). Trastuzumab deruxtecan in previously treated HER2-low advanced breast cancer. N. Engl. J. Med..

[7] Mosele F., Deluche E., Lusque A., Le Bescond L., Filleron T., Pradat Y., Ducoulombier A., Pistilli B., Bachelot T., Viret F. (2023). Trastuzumab deruxtecan in metastatic breast cancer with variable HER2 expression: The phase 2 DAISY trial. Nat. Med..

[8] Bardia A., Hu X., Dent R., Yonemori K., Barrios C.H., O’Shaughnessy J.A., Wildiers H., Pierga J.Y., Zhang Q., Saura C. (2024). Trastuzumab deruxtecan after endocrine therapy in metastatic breast cancer. N. Engl. J. Med..

[9] American College of Pathologists Reporting Template for Reporting Results of Biomarker Testing of Specimens from Patients with Carcinoma of the Breast. https://documents.cap.org/documents/New-Cancer-Protocols-March-2025/Breast.Bmk_1.6.0.0.REL.CAPCP.pdf.

[10] Daiichi Sankyo Inc (2025). ENHERTU^®^ Approved in the EU as First HER2 Directed Therapy for Patients with HR Positive, HER2 Low or HER2 Ultralow Metastatic Breast Cancer Following at Least One Endocrine Therapy. [Press Release]. 4 April 2025. https://www.daiichisankyo.com/files/news/pressrelease/pdf/202504/20250404_E.pdf.

[11] Wolff A.C., Hammond M.E., Hicks D.G., Dowsett M., McShane L.M., Allison K.H., Allred D.C., Bartlett J.M., Bilous M., Fitzgibbons P. (2013). Recommendations for human epidermal growth factor receptor 2 testing in breast cancer: American Society of Clinical Oncology/College of American Pathologists clinical practice guideline update. J. Clin. Oncol..

[12] Morales S., Gasol A., Sanchez D.R. (2021). Her2-positive cancers and antibody-based treatment: State of the art and future developments. Cancers.

[13] Tarantino P., Hamilton E., Tolaney S.M., Cortes J., Morganti S., Ferraro E., Marra A., Viale G., Trapani D., Cardoso F. (2020). HER2-low breast cancer: Pathological and clinical landscape. J. Clin. Oncol..

[14] Prat A., Bardia A., Curigliano G., Hammond M.E.H., Loibl S., Tolaney S.M., Viale G. (2022). An overview of clinical development of agents for metastatic or advanced breast cancer without ERBB2 amplification (HER2-Low). JAMA Oncol..

[15] Chen Z., Jia H., Zhang H., Chen L., Zhao P., Zhao J., Fu G., Xing X., Li Y., Wang C. (2023). Is HER2 ultra-low breast cancer different from HER2 null or HER2 low breast cancer? A study of 1363 patients. Breast Cancer Res. Treat..

[16] Viale G., Basik M., Niikura N., Tokunaga E., Brucker S., Penault-Llorca F., Hayashi N., Sohn J., Teixeira de Sousa R., Brufsky A.M. (2023). Retrospective study to estimate the prevalence and describe the clinicopathological characteristics, treatments received, and outcomes of HER2-low breast cancer. ESMO Open.

[17] Baez-Navarro X., van Bockstal M.R., Nawawi D., Broeckx G., Colpaert C., Doebar S.C., Hogenes M.C.H., Koop E., Lambein K., Peeters D.J.E. (2023). Interobserver variation in the assessment of immunohistochemistry expression levels in HER2-negative breast cancer: Can we improve the identification of low levels of HER2 expression by adjusting the criteria? An international interobserver study. Mod. Pathol..

[18] Curigliano G., Dent R., Earle H., Modi S., Tarantino P., Viale G., Tolaney S.M. (2024). Open questions, current challenges, and future perspectives in targeting human epidermal growth factor receptor 2-low breast cancer. ESMO Open.

[19] Kang S., Lee S.H., Lee H.J., Jeong H., Jeong J.H., Kim J.E., Ahn J.H., Jung K.H., Gong G., Kim H.H. (2022). Pathological complete response, long-term outcomes, and recurrence patterns in HER2-low versus HER2-zero breast cancer after neoadjuvant chemotherapy. Eur. J. Cancer.

[20] Peiffer D.S., Zhao F., Chen N., Hahn O.M., Nanda R., Olopade O.I., Huo D., Howard F.M. (2023). Clinicopathologic characteristics and prognosis of ERBB2-low breast cancer among patients in the national cancer database. JAMA Oncol..

[21] Bae S.Y., Kim S., Lee J.H., Lee H.C., Lee S.K., Kil W.H., Kim S.W., Lee J.E., Nam S.J. (2015). Poor prognosis of single hormone receptor- positive breast cancer: Similar outcome as triple-negative breast cancer. BMC Cancer.

[22] Schettini F., Chic N., Brasó-Maristany F., Paré L., Pascual T., Conte B., Martínez-Sáez O., Adamo B., Vidal M., Barnadas E. (2021). Clinical, pathological, and PAM50 gene expression features of HER2-low breast cancer. NPJ Breast Cancer.

[23] Miglietta F., Griguolo G., Bottosso M., Giarratano T., Lo Mele M., Fassan M., Cacciatore M., Genovesi E., De Bartolo D., Vernaci G. (2021). Evolution of HER2-low expression from primary to recurrent breast cancer. NPJ Breast Cancer.

[24] Matikas A., Foukakis T., Bergh J. (2017). Tackling endocrine resistance in ER-positive HER2-negative advanced breast cancer: A tale of imprecision medicine. Crit. Rev. Oncol. Hematol..

[25] Zhang H., Peng Y. (2022). Current biological, pathological and clinical landscape of HER2-low breast cancer. Cancers.

[26] Ferrari P., Scatena C., Ghilli M., Bargagna I., Lorenzini G., Nicolini A. (2022). Molecular mechanisms, biomarkers and emerging therapies for chemotherapy resistant TNBC. Int. J. Mol. Sci..

[27] European Society for Medical Oncology ESMO Metastatic Breast Cancer Living Guidelines, v1.1. https://www.esmo.org/living-guidelines/esmo-metastatic-breast-cancer-living-guideline.

[28] Gennari A., André F., Barrios C.H., Cortés J., de Azambuja E., DeMichele A., Dent R., Fenlon D., Gligorov J., Hurvitz S.A. (2021). ESMO Clinical Practice Guideline for the diagnosis, staging and treatment of patients with metastatic breast cancer. Ann. Oncol..

[B29-cancers-17-04021] 29.Referenced from the NCCN Clinical Practice Guidelines in Oncology (NCCN Guidelines®) for Breast Cancer V.5.2025. © National Comprehensive Cancer Network, Inc, 2025. All rights reserved. Available online: https://www.nccn.org/guidelines/guidelines-detail?category=1&id=1419 (accessed 3 December 2025). To view the most recent and complete version of the guideline, go online to NCCN.org. NCCN makes no warranties of any kind whatsoever regarding their content, use or application and disclaims any responsibility for their application or use in any way.

[30] Andrahennadi S., Sami A., Manna M., Pauls M., Ahmed S. (2021). Current landscape of targeted therapy in hormone receptor-positive and HER2-negative breast cancer. Curr. Oncol..

[31] Yang C., Brezden-Masley C., Joy A.A., Sehdev S., Modi S., Simmons C., Henning J.W. (2023). Targeting HER2-low in metastatic breast cancer: An evolving treatment paradigm. Ther. Adv. Med. Oncol..

[32] Yamamoto Y., Yamauchi C., Toyama T., Nagai S., Sakai T., Kutomi G., Yoshimura M., Kawai M., Ohtani S., Kubota K. (2024). The Japanese Breast Cancer Society Clinical Practice Guidelines for Breast Cancer, 2022 Edition: Changes from the 2018 edition and general statements on breast cancer treatment. Breast Cancer.

[33] Slamon D.J., Neven P., Chia S., Fasching P.A., De Laurentiis M., Im S.A., Petrakova K., Bianchi G.V., Esteva F.J., Martín M. (2018). Phase III randomized study of ribociclib and fulvestrant in hormone receptor-positive, human epidermal growth factor receptor 2-negative advanced breast cancer: MONALEESA-3. J. Clin. Oncol..

[34] Llombart-Cussac A., Harper-Wynne C., Perello A., Hennequin A., Fernandez A., Colleoni M., Carañana V., Quiroga V., Medioni J., Iranzo V. (2023). Second-line endocrine therapy (ET) with or without palbociclib (P) maintenance in patients (pts) with hormone receptor-positive (HR[+])/human epidermal growth factor receptor 2-negative (HER2[-]) advanced breast cancer (ABC): PALMIRA trial. J. Clin. Oncol..

[35] Kalinsky K., Accordino M.K., Chiuzan C., Mundi P.S., Trivedi M.S., Novik Y., Tiersten A., Raptis G., Baer L.N., Young Oh S. (2022). A randomized, phase II trial of fulvestrant or exemestane with or without ribociclib after progression on anti-estrogen therapy plus cyclin-dependent kinase 4/6 inhibition (CDK 4/6i) in patients (pts) with unresectable or hormone receptor–positive (HR+), HER2-negative metastatic breast cancer (MBC): MAINTAIN trial. J. Clin. Oncol..

[36] Mayer E.L., Ren Y., Wagle N., Mahtani R., Ma C., DeMichele A., Cristofanilli M., Meisel J.L., Miller K.D., Jolly T. (2023). Abstract GS3-06: GS3-06 palbociclib after CDK4/6i and endocrine therapy (PACE): A randomized phase II study of fulvestrant, palbociclib, and avelumab for endocrine pre-treated ER+/HER2- metastatic breast cancer. Cancer Res..

[37] Dickler M.N., Tolaney S.M., Rugo H.S., Cortés J., Diéras V., Patt D., Wildiers H., Hudis C.A., O’Shaughnessy J., Zamora E. (2017). MONARCH 1, A phase II study of abemaciclib, a CDK4 and CDK6 inhibitor, as a single agent, in patients with refractory HR(+)/HER2(−) metastatic breast cancer. Clin. Cancer Res..

[38] O’Sullivan C.C., Clarke R., Goetz M.P., Robertson J. (2023). Cyclin-dependent kinase 4/6 inhibitors for treatment of hormone receptor-positive, ERBB2-negative breast cancer: A review. JAMA Oncol..

[39] Nuzzolese I., Montemurro F. (2020). Attrition in metastatic breast cancer: A metric to be reported in randomised clinical trials?. Lancet Oncol..

[40] Hortobagyi G.N., Stemmer S.M., Burris H.A., Yap Y.S., Sonke G.S., Paluch-Shimon S., Campone M., Petrakova K., Blackwell K.L., Winer E.P. (2018). Updated results from MONALEESA-2, a phase III trial of first-line ribociclib plus letrozole versus placebo plus letrozole in hormone receptor-positive, HER2-negative advanced breast cancer. Ann. Oncol..

[41] Tripathy D., Im S.A., Colleoni M., Franke F., Bardia A., Harbeck N., Hurvitz S.A., Chow L., Sohn J., Lee K.S. (2018). Ribociclib plus endocrine therapy for premenopausal women with hormone-receptor-positive, advanced breast cancer (MONALEESA-7): A randomised phase 3 trial. Lancet Oncol..

[42] Rugo H.S., Finn R.S., Diéras V., Ettl J., Lipatov O., Joy A.A., Harbeck N., Castrellon A., Iyer S., Lu D.R. (2019). Palbociclib plus letrozole as first-line therapy in estrogen receptor-positive/human epidermal growth factor receptor 2-negative advanced breast cancer with extended follow-up. Breast Cancer Res. Treat..

[43] Johnston S., Martin M., Di Leo A., Im S.A., Awada A., Forrester T., Frenzel M., Hardebeck M.C., Cox J., Barriga S. (2019). MONARCH 3 final PFS: A randomized study of abemaciclib as initial therapy for advanced breast cancer. NPJ Breast Cancer.

[44] Hartkopf A.D., Walter C.B., Kolberg H.C., Hadji P., Tesch H., Fasching P.A., Ettl J., Lüftner D., Wallwiener M., Müller V. (2024). Attrition in the first three therapy lines in patients with advanced breast cancer in the german real-world PRAEGNANT Registry. Geburtshilfe Frauenheilkd.

[45] Rugo H.S., Brufsky A., Liu X., Li B., McRoy L., Chen C., Layman R.M., Cristofanilli M., Torres M.A., Curigliano G. (2022). Real-world study of overall survival with palbociclib plus aromatase inhibitor in HR+/HER2- metastatic breast cancer. NPJ Breast Cancer.

[46] Twelves C., Cheeseman S., Sopwith W., Thompson M., Riaz M., Ahat-Donker N., Myland M., Lee A., Przybysz R., Turner S. (2020). Systemic treatment of hormone receptor positive, human epidermal growth factor 2 negative metastatic breast cancer: Retrospective analysis from Leeds Cancer Centre. BMC Cancer.

[47] Morikawa A., Seidman A.D. (2015). Treating triple-negative breast cancer: Where are we?. J. Natl. Comp. Canc. Netw..

[48] Chen N., Matossian M., Saha P., Rampurwala M., Kamaraju S., Hahn O., Howard F.M., Flemming G.F., Freeman J.Q., Karrison T. (2025). A randomized pahse II trial of nab-paclitaxel with or without mifepristone for advanced triple-negative breast cancer. Breast Cancer Res. Treat..

[49] Tolaney S.M., de Azambuja E., Kalinsky K., Loi S., Kim S.-B., Yam C., Rapoport B.L., Im S.-A., Pistilli B., McHayleh W. (2025). Sacituzumab govitecan (SG) + pembrolizumab (pembro) vs chemotherapy (chemo) + pembro in previously untreated PD-L1–positive advanced triple-negative breast cancer (TNBC): Primary results from the randomized phase 3 ASCENT-04/KEYNOTE-D19 study. J. Clin. Oncol..

[50] Cortes J., Cescon D.W., Rugo H.S., Nowecki Z., Im S.A., Yusof M.M., Gallardo C., Lipatov O., Barrios C.H., Holgado E. (2020). Pembrolizumab plus chemotherapy versus placebo plus chemotherapy for previously untreated locally recurrent inoperable or metastatic triple-negative breast cancer (KEYNOTE-355): A randomised, placebo-controlled, double-blind, phase 3 clinical trial. Lancet.

[51] Ogitani Y., Aida T., Hagihara K., Yamaguchi J., Ishii C., Harada N., Soma M., Okamoto H., Oitate M., Arakawa S. (2016). DS-8201a, a novel HER2-targeting ADC with a novel DNA topoisomerase I inhibitor, demonstrates a promising antitumor efficacy with differentiation from T-DM1. Clin. Cancer Res..

[52] Ogitani Y., Hagihara K., Oitate M., Naito H., Agatsuma T. (2016). Bystander killing effect of DS-8201a, a novel anti-human epidermal growth factor receptor 2 antibody-drug conjugate, in tumors with human epidermal growth factor receptor 2 heterogeneity. Cancer Sci..

[53] Nakada T., Sugihara K., Jikoh T., Abe Y., Agatsuma T. (2019). The latest research and development into the antibody-drug conjugate, [fam-] trastuzumab deruxtecan (DS-8201a), for HER2 cancer therapy. Chem. Pharm. Bull..

[54] Han S., Lim K.S., Blackburn B.J., Yun J., Putnam C.W., Bull D.A., Won Y.W. (2022). The potential of topoisomerase inhibitor-based antibody-drug conjugates. Pharmaceutics.

[55] Tsao L.C., Wang J.S., Ma X., Sodhi S., Ragusa J.V., Liu B., McBane J., Wang T., Wei J., Liu C.X. (2025). Effective extracellular payload release and immunomodulatory interactions govern the therapeutic effect of trastuzumab deruxtecan (T-DXd). Nat. Commun..

[56] Modi S., Jacot W., Iwata H., Park Y.H., Vidal Losada M., Li W., Tsurutani J., Ueno N.T., Zaman K., Prat A. (2025). Trastuzumab deruxtecan in HER2-low metastatic breast cancer: Long-term survival analysis of the randomized, phase 3 DESTINY-Breast04 trial. Nat. Med..

[57] Daiichi Sankyo Inc (2025). ENHERTU^®^ (Fam-Trastuzumab Deruxtecan-Nxki) for Injection, for Intravenous Use. https://www.accessdata.fda.gov/drugsatfda_docs/label/2024/761139s028lbl.pdf.

[58] Gilead Sciences (2023). TRODELVY^®^ (Sacituzumab Govitecan-Hziy) for Injection, for Intravenous Use. https://www.accessdata.fda.gov/drugsatfda_docs/label/2023/761115s035lbl.pdf.

[59] Rugo H.S., Bardia A., Marmé F., Cortes J., Schmid P., Loirat D., Trédan O., Ciruelos E., Dalenc F., Pardo P.G. (2022). Sacituzumab govitecan in hormone receptor-positive/human epidermal growth factor receptor 2-negative metastatic breast cancer. J. Clin. Oncol..

[60] Rugo H.S., Bardia A., Marmé F., Cortés J., Schmid P., Loirat D., Trédan O., Ciruelos E., Dalenc F., Gómez Pardo P. (2023). Overall survival with sacituzumab govitecan in hormone receptor-positive and human epidermal growth factor receptor 2-negative metastatic breast cancer (TROPiCS-02): A randomised, open-label, multicentre, phase 3 trial. Lancet.

[61] Bardia A., Hurvitz S.A., Tolaney S.M., Loirat D., Punie K., Oliveira M., Brufsky A., Sardesai S.D., Kalinsky K., Zelnak A.B. (2021). Sacituzumab govitecan in metastatic triple-negative breast cancer. N. Engl. J. Med..

[62] Daiichi Sankyo, Inc (2025). DATROWAY^®^ (Datopotamab Deruxtecan-Dlnk) for Injection, for Intravenous Use. https://www.accessdata.fda.gov/drugsatfda_docs/label/2025/761394s000lbl.pdf.

[63] Bardia A., Jhaveri K., Im S.A., Pernas S., De Laurentiis M., Wang S., Martínez Jañez N., Borges G., Cescon D.W., Hattori M. (2024). Datopotamab deruxtecan versus chemotherapy in previously treated inoperable/metastatic hormone receptor-positive human epidermal growth factor receptor 2-negative breast cancer: Primary results from TROPION-Breast01. J. Clin. Oncol..

[64] Pistilli B., Jhaveri K., Im S.-A., Pernas Simon S.P., De Laurentiis M., Wang S., Martinez N., Santos Borges G., Cescon D., Hattori M. (2025). Datopotamab deruxtecan (Dato-DXd) vs chemotherapy (CT) in previously-treated inoperable or metastatic hormone receptor-positive, HER2-negative (HR+/HER2–) breast cancer (BC): Final overall survival (OS) from the phase III TROPION-Breast01 trial. Ann. Oncol..

[65] Chen M., Huang R., Chen R., Pan F., Shen X., Li H., Rong Q., An X., Xue C., Shi Y. (2024). Optimal sequential strategies for antibody-drug conjugate in metastatic breast cancer: Evaluating efficacy and cross-resistance. Oncologist.

[66] Abelman R.O., Spring L., Fell G.G., Ryan P., Vidula N., Medford A.J., Shin J., Abraham E., Wander S.A., Insakoff S.J. (2024). Sequential use of antibody-drug conjugate after antibody-drug conjugate for patients with metastatic breast cancer: ADC after ADC (A3) study. J. Clin. Oncol..

[67] Mai N., Klar M., Ferraro E., Bromberg M., Chen Y., Razavi P., Modi S., Chandarlapaty S., Walsh E.M., Drago J.Z. (2024). Real world outcomes of sequential ADC therapy in metastatic breast cancer: Patients treated with sacituzumab govitecan and trastuzumab deruxtecan. J. Clin. Oncol..

[68] Poumeaud F., Morisseau M., Cabel L., Gonçalves A., Rivier C., Trédan O., Volant E., Frenel J.S., Ladoire S., Jacot W. (2024). Efficacy of administration sequence: Sacituzumab Govitecan and Trastuzumab Deruxtecan in HER2-low metastatic breast cancer. Br. J. Can..

[69] Huppert L.A., Mahtani R., Fisch S., Dempsey N., Premji S., Raimonde A., Jacob S., Quintal L., Melisko M., Chien J. (2025). Multicenter retrospective cohort study of the sequential use of the antibody-drug conjugates (ADCs) trastuzumab deruxtecan (T-DXd) and sacituzumab govitecan (SG) in patients with HER2-low metastatic breast cancer (MBC). NPJ Breast Cancer.

[70] Collins D.M., Bossenmaier B., Kollmorgen G., Niederfellner G. (2019). Acquired resistance to antibody-drug conjugates. Cancers.

[71] Abelman R.O., Spring L., Fell G., Davis A., Hensing W., Ryan P., Vidula N., Wander S., Medford A., Shin J. (2024). Sequencing antibody-drug conjugate after antibody-drug conjugate in metastatic breast cancer (A3 study): Multi-institution experience and biomarker analysis. Cancer Res..

[72] Fenton M.A., Tarantino P., Graff S.L. (2023). Sequencing antibody drug conjugates in breast cancer: Exploring future roles. Curr. Oncol..

[73] Saltalamacchia G., Torrisi R., De Sanctis R., Masci G., Miggiano C., Gaudio M., Benvenuti C., Jacobs F., Gerosa R., Santoro A. (2024). Charting the course in sequencing antibody-drug conjugates in breast cancer. Biomedicines.

[74] Guven D.C., Kaya M.B., Fedai B., Ozden M., Yildirim H.C., Kosemehmetoglu K., Kertmen N., Dizdar O., Uner A., Aksoy S. (2022). HER2-low breast cancer could be associated with an increased risk of brain metastasis. Int. J. Clin. Oncol..

[75] Jin J., Li B., Cao J., Li T., Zhang J., Cao J., Zhao M., Wang L., Wang B., Tao Z. (2023). Analysis of clinical features, genomic landscapes and survival outcomes in HER2-low breast cancer. J. Transl. Med..

[76] Harbeck N., Modi S., Jacot W., Yamashita T., Sohn J., Vidal M., Tsurutani J., Ueno N.T., Prat A., Niikura N. (2022). Trastuzumab deruxtecan vs treatment of physician’s choice in patients with HER2-low unresectable and/or metastatic breast cancer: Subgroup analyses from DESTINY-Breast04. Cancer Res..

[77] Tsurutani J., Jacot W., Yamashita T., Riaz F., Yerushalmi R., Im S.-A., Niikura N., Halser-Strub U., Cortés J., Wennstig A.-K. (2023). Subgroup analysis of patients (pts) with HER2-low metastatic breast cancer (mBC) with brain metastases (BMs) at baseline from DESTINY-Breast04, a randomized phase III study of trastuzumab deruxtecan (T-DXd) vs treatment of physician’s choice (TPC). Ann. Oncol..

[78] Vaz Batista M., Pérez-García J.M., Cortez P., Garrigós L., Fernández-Abad M., Gion M., Martínez-Bueno A., Saavedra C., Teruel I., Fernandez-Ortega A. (2024). Trastuzumab deruxtecan in patients with previously treated HER2-low advanced breast cancer and active brain metastases: The DEBBRAH trial. ESMO Open.

[79] Hechtman J.F., Ross D.S. (2019). The past, present, and future of HER2 (ERBB2) in cancer: Approaches to molecular testing and an evolving role in targeted therapy. Cancer Cytopathol..

[80] Tozbikian G., Bui M.M., Hicks D.G., Jaffer S., Khoury T., Wen H.Y., Krishnamurthy S., Wei S. (2024). Best practices for achieving consensus in HER2-low expression in breast cancer: Current perspectives from practising pathologists. Histopathology.

[81] Pauletti G., Godolphin W., Press M.F., Slamon D.J. (1996). Detection and quantitation of HER-2/neu gene amplification in human breast cancer archival material using fluorescence in situ hybridization. Oncogene.

[82] Marchiò C., Annaratone L., Marques A., Casorzo L., Berrino E., Sapino A. (2021). Evolving concepts in HER2 evaluation in breast cancer: Heterogeneity, HER2-low carcinomas and beyond. Semin. Cancer Biol..

[83] Garrido C., Manoogian M., Ghambire D., Lucas S., Karnoub M., Olson M.T., Hicks D.G., Tozbikian G., Prat A., Ueno N.T. (2024). Analytical and clinical validation of PATHWAY Anti-HER-2/neu (4B5) antibody to assess HER2-low status for trastuzumab deruxtecan treatment in breast cancer. Virchows Arch..

[84] Hoffman La Roche Ltd (2024). Roche Obtains CE Mark for First Companion Diagnostic to Identify Patients with HER2-Low Metastatic Breast Cancer Eligible for ENHERTU. [Press Release]. 10 April 2024. https://diagnostics.roche.com/global/en/news-listing/2024/roche-obtains-ce-mark-for-first-companion-diagnostic-to-identify-patients-with-her2-low-metastatic-breast-cancer-eligible-for-enhertu.html.

[85] Baez-Navarro X., Salgado R., Denkert C., Lennerz J.K., Penault-Llorca F., Viale G., Bartlett J.M.S., van Deurzen C.H.M. (2022). Selecting patients with HER2-low breast cancer: Getting out of the tangle. Eur. J. Cancer.

[86] Ivanova M., Porta F.M., D’Ercole M., Pescia C., Sajjadi E., Cursano G., De Camilli E., Pala O., Mazzarol G., Venetis K. (2024). Standardized pathology report for HER2 testing in compliance with 2023 ASCO/CAP updates and 2023 ESMO consensus statements on HER2-low breast cancer. Virchows Arch..

[87] Sajjadi E., Guerini-Rocco E., De Camilli E., Pala O., Mazzarol G., Venetis K., Ivanova M., Fusco N. (2023). Pathological identification of HER2-low breast cancer: Tips, tricks, and troubleshooting for the optimal test. Front. Mol. Biosci..

[88] Fernandez A.I., Liu M., Bellizzi A., Brock J., Fadare O., Hanley K., Harigopal M., Jorns J.M., Kuba M.G., Ly A. (2022). Examination of low ERBB2 protein expression in breast cancer tissue. JAMA Oncol..

[89] Zoppoli G., Garuti A., Cirmena G., di Cantogno L.V., Botta C., Gallo M., Ferraioli D., Carminati E., Baccini P., Curto M. (2017). Her2 assessment using quantitative reverse transcriptase polymerase chain reaction reliably identifies Her2 overexpression without amplification in breast cancer cases. J. Transl. Med..

[90] Seung B.J., Cho S.H., Kim S.H., Lim H.Y., Sur J.H. (2020). Quantitative analysis of HER2 mRNA expression by RNA in situ hybridization in canine mammary gland tumors: Comparison with immunohistochemistry analysis. PLoS ONE.

[91] Moutafi M., Robbins C.J., Yaghoobi V., Fernandez A.I., Martinez-Morilla S., Xirou V., Bai Y., Song Y., Gaule P., Krueger J. (2022). Quantitative measurement of HER2 expression to subclassify ERBB2 unamplified breast cancer. Lab. Investig..

[92] Hicks D.G., Buscaglia B., Goda H., McMahon L., Natori T., Turner B., Soukiazian A., Okada H., Nakano Y. (2018). A novel detection methodology for HER2 protein quantitation in formalin-fixed, paraffin embedded clinical samples using fluorescent nanoparticles: An analytical and clinical validation study. BMC Cancer.

[93] Wu S., Yue M., Zhang J., Li X., Li Z., Zhang H., Wang X., Han X., Cai L., Shang J. (2023). The role of artificial intelligence in accurate interpretation of HER2 immunohistochemical scores 0 and 1+ in breast cancer. Mod. Pathol..

[94] Goldsmith J.D., Troxell M.L., Roy-Chowdhuri S., Colasacco C.F., Edgerton M.E., Fitzgibbons P.L., Fulton R., Haas T., Kandalaft P.L., Kalicanin T. (2024). Principles of Analytic Validation of Immunohistochemical Assays: Guideline Update. Arch. Pathol. Lab. Med..

[95] US Food and Drug Administration (2025). FDA Approves Fam-Trastuzumab Deruxtecan-Nxki for Unresectable or Metastatic HR-Positive, HER2-Low or HER2-Ultralow Breast Cancer. [Press Release]. 6 February 2025. https://www.fda.gov/drugs/resources-information-approved-drugs/fda-approves-fam-trastuzumab-deruxtecan-nxki-unresectable-or-metastatic-hr-positive-her2-low-or-her2.

[96] Nicolò E., Boscolo Bielo L., Curigliano G., Tarantino P. (2023). The HER2-low revolution in breast oncology: Steps forward and emerging challenges. Ther. Adv. Med. Oncol..

[97] Scott M., Vandenberghe M.E., Scorer P., Boothman A.-M., Barker C. (2021). Prevalence of HER2 low in breast cancer subtypes using the VENTANA anti-HER2/neu (4B5) assay. J. Clin. Oncol..

[98] Sauter G., Lee J., Bartlett J.M., Slamon D.J., Press M.F. (2009). Guidelines for human epidermal growth factor receptor 2 testing: Biologic and methodologic considerations. J. Clin. Oncol..

[99] Rüschoff J., Friedrich M., Nagelmeier I., Kirchner M., Andresen L.M., Salomon K., Portier B., Sredni S.T., Schildhaus H.U., Jasani B. (2022). Comparison of HercepTest™ mAb pharmDx (Dako Omnis, GE001) with Ventana PATHWAY anti-HER-2/neu (4B5) in breast cancer: Correlation with HER2 amplification and HER2 low status. Virchows Arch..

[100] Schildhaus H.-U., Badve S., D’Arrigo C., Farshid G., Lebeau A., Peg V., Penault-Llorca F., Rueschoff J., Yang W., Atkey N. (2024). Concordance between the DESTINY-Breast04 clinical trial assay (4B5[CDx]) and other HER2 IHC assays for HER2-low breast cancer in real-world practice: First phase of a large-scale, multicenter global ring study. Cancer Res..

[101] Krishnamurthy S., Lam C., Luo L., Baverstock R., Pyrih N., Redpath S., Sue-Ann Woo M., Collin S.M., Davies C., Gannon V. (2025). Abstract P3-09-20: Re-evaluation of human epidermal growth factor receptor 2 (HER2) immunohistochemistry (IHC) 0 or 1+ in metastatic breast cancer (mBC) samples to characterize the proportion of HER2-ultralow (IHC 0 with membrane staining). Clin. Cancer Res..

[102] Hanna W.M., Rüschoff J., Bilous M., Coudry R.A., Dowsett M., Osamura R.Y., Penault-Llorca F., van de Vijver M., Viale G. (2014). HER2 in situ hybridization in breast cancer: Clinical implications of polysomy 17 and genetic heterogeneity. Mod. Pathol..

[103] Bergeron A., Bertaut A., Beltjens F., Charon-Barra C., Amet A., Jankowski C., Desmoulins I., Ladoire S., Arnould L. (2023). Anticipating changes in the HER2 status of breast tumours with disease progression-towards better treatment decisions in the new era of HER2-low breast cancers. Br. J. Cancer.

[104] Bar Y., Dedeoglu A.S., Fell G.G., Moffett N.J., Boyraz B., Ly A., Bardia A., Moy B., Ellisen L.W., Isakoff S.J. (2023). Dynamic HER2-low status among patients with triple negative breast cancer (TNBC): The impact of repeat biopsies. J. Clin. Oncol..

[105] Hou Y., Nitta H., Li Z. (2023). HER2 intratumoral heterogeneity in breast cancer, an evolving concept. Cancers.

[106] Geukens T., De Schepper M., Richard F., Maetens M., Van Baelen K., Mahdami A., Nguyen H.L., Isnaldi E., Leduc S., Pabba A. (2023). Intra-patient and inter-metastasis heterogeneity of HER2-low status in metastatic breast cancer. Eur. J. Cancer.

[107] Tarantino P., Gandini S., Nicolò E., Trillo P., Giugliano F., Zagami P., Vivanet G., Bellerba F., Trapani D., Marra A. (2022). Evolution of low HER2 expression between early and advanced-stage breast cancer. Eur. J. Cancer.

[108] Löb S., Linsmeier E., Herbert S.L., Schlaiß T., Kiesel M., Wischhusen J., Salmen J., Kranke P., Quenzer A., Kurz F. (2023). Prognostic effect of HER2 evolution from primary breast cancer to breast cancer metastases. J. Cancer Res. Clin. Oncol..

[109] Lin M., Luo T., Jin Y., Zhong X., Zheng D., Zeng C., Guo Q., Wu J., Shao Z.M., Hu X. (2024). HER2-low heterogeneity between primary and paired recurrent/metastatic breast cancer: Implications in treatment and prognosis. Cancer.

[110] Holthuis E.I., Vondeling G.T., Kuiper J.G., Dezentjé V., Rosenlund M., Overbeek J.A., van Deurzen C.H.M. (2022). Real-world data of HER2-low metastatic breast cancer: A population based cohort study. Breast.

[111] de Calbiac O., Lusque A., Mailliez A., Bachelot T., Uwer L., Mouret-Reynier M.A., Emile G., Jouannaud C., Gonçalves A., Patsouris A. (2022). Comparison of management and outcomes in ERBB2-low vs ERBB2-Zero metastatic breast cancer in France. JAMA Netw. Open.

[112] Garrido-Castro A.C., Ngo L.D., Richardson E.T., Frangieh A., Mohammed-Abreu A., Hughes M.E., Zanudo J.G.T., Navarro J., Tarantino P., Mittendorf E.A. (2023). Abstract HER2-10: HER2-10 Dynamics of HER2-low expression in triple-negative breast cancer. Cancer Res..

[113] Alvarez Lopez I., Guerrero Zotano A.L., Antolín-Novoa S., Tibau A., Falo C., Hernández Sosa M., Miguel Rodriguez A., Rodríguez-Lescure A., Martinez del Prado P., Chacon J.I. (2023). Characteristics and outcomes of HER2-low (H-Low) and HER2-zero (H-0) advanced breast cancer (ABC) patients (pts) from GEICAM/2014-03 (RegistEM) registry. ESMO Breast Cancer.

[114] Hong B., Ying F., Zhaoqing F., Xichun H., Man L., Qiao L., Ning L., Ting L., Jianyun N., Yueyin P. (2023). Consensus on clinical diagnosis and medical treatment of HER2-low breast cancer (2022 edition). J. Natl. Cancer Cent..

[115] Geukens T., De Schepper M., Richard F., Maetens M., Van Baelen K., Mahdami A., Nguyen H.-L., Isnaldi E., Leduc S., Pabba A. (2023). Abstract HER2-16: HER2-16 Inter-lesion heterogeneity of HER2-status in metastatic breast cancer: Possible implications for treatment with anti-HER2 antibody-drug conjugates. Cancer Res..

[116] Ueno N.T., Jacot W., Yamashita T., Sohn J., Tokunaga E., Prat A., Tsurutani J., Park Y.H., Rugo H.S., Xu B. (2022). Patient-reported outcomes (PROs) from DESTINY-Breast04, a randomized phase III study of trastuzumab deruxtecan (T-DXd) vs treatment of physician’s choice (TPC) in patients (pts) with HER2-low metastatic breast cancer (MBC). Ann. Oncol..

[117] Hu X., Curigliano G., Yonemori K., Bardia A., Barrios C.H.E., Sohn J., Levy C., Jacot W., Tsurutani J., K. M. (2024). Effects of trastuzumab deruxtecan (T-DXd) vs choice of chemotherapy (TPC) on patient-reported outcomes (PROs) in hormone receptor-positive, HER2-low or HER2-ultralow metastatic breast cancer (mBC): Results from DESTINY-Breast06. ESMO Congr..

[118] Ueno N.T., Cottone F., Dunton K., Jacot W., Yamashita T., Sohn J., Tokunaga E., Prat A., Tsurutani J., Park Y.H. (2025). Patient-reported outcomes from DESTINY-Breast04: Trastuzumab deruxtecan versus physician’s choice of chemotherapy in patients with HER2-low mBC. Oncologist.

[119] Rugo H.S., Bianchini G., Cortes J., Henning J.W., Untch M. (2022). Optimizing treatment management of trastuzumab deruxtecan in clinical practice of breast cancer. ESMO Open.

[120] Abuhelwa Z., Alloghbi A., Alqahtani A., Nagasaka M. (2022). Trastuzumab deruxtecan-induced interstitial lung disease/pneumonitis in ERBB2-positive advanced solid malignancies: A systematic review. Drugs.

[121] Swain S.M., Nishino M., Lancaster L.H., Li B.T., Nicholson A.G., Bartholmai B.J., Naidoo J., Schumacher-Wulf E., Shitara K., Tsurutani J. (2022). Multidisciplinary clinical guidance on trastuzumab deruxtecan (T-DXd)–related interstitial lung disease/pneumonitis—Focus on proactive monitoring, diagnosis, and management. Cancer Treat. Rev..

[122] Powell C.A., Modi S., Iwata H., Takahashi S., Smit E.F., Siena S., Chang D.Y., Macpherson E., Qin A., Singh J. (2022). Pooled analysis of drug-related interstitial lung disease and/or pneumonitis in nine trastuzumab deruxtecan monotherapy studies. ESMO Open.

[123] Park Y.H., Jacot W., Hurvitz S.A., Modi S., Yamashita T., Xu B., Tokunaga E., Wang X., Lee K.S., Iwata H. (2024). Exploratory pooled safety analysis of trastuzumab deruxtecan (T-DXd) in patients With HER2+ or HER2-low unresectable and/or metastatic breast cancer (mBC) in DESTINY-Breast trials. ESMO Open.

[124] Ciruelos E., García-Sáenz J., Gavilá J., Martín M., Rodríguez C.A., Rodríguez-Lescure Á. (2024). Safety profile of trastuzumab deruxtecan in advanced breast cancer: Expert opinion on adverse event management. Clin. Transl. Oncol..

[125] Skeoch S., Weatherley N., Swift A.J., Oldroyd A., Johns C., Hayton C., Giollo A., Wild J.M., Waterton J.C., Buch M. (2018). Drug-induced interstitial lung disease: A systematic review. J. Clin. Med..

[126] Johkoh T., Lee K.S., Nishino M., Travis W.D., Ryu J.H., Lee H.Y., Ryerson C.J., Franquet T., Bankier A.A., Brown K.K. (2021). Chest CT diagnosis and clinical management of drug-related pneumonitis in patients receiving molecular targeting agents and immune checkpoint inhibitors: A position paper from the Fleischner Society. Radiology.

[127] Wekking D., Porcu M., Pellegrino B., Lai E., Mura G., Denaro N., Saba L., Musolino A., Scartozzi M., Solinas C. (2023). Multidisciplinary clinical guidelines in proactive monitoring, early diagnosis, and effective management of trastuzumab deruxtecan (T-DXd)-induced interstitial lung disease (ILD) in breast cancer patients. ESMO Open.

[128] Tarantino P., Tolaney S.M. (2023). Detecting and managing T-DXd-related interstitial lung disease: The five “S” rules. JCO Oncol. Pract..

[129] Rugo H.S., Crossno C.L., Gesthalter Y.B., Kelley K., Moore H.B., Rimawi M.F., Westbrook K.E., Buys S.S. (2023). Real-world perspectives and practices for pneumonitis/interstitial lung disease associated with trastuzumab deruxtecan use in human epidermal growth factor receptor 2-expressing metastatic breast cancer. JCO Oncol. Pract..

[130] Natsuhara K.H., Blum K., LeVee A.A., Vobugari N., Dempsey N., Premji S.K., Moe C., Reddy N., Hoppenworth J.E., Vella M. (2025). Treatment rechallenge after trastuzumab-deruxtecan–related interstitial lung disease: A multi-institution cohort study. J. Clin. Oncol..

[B131-cancers-17-04021] 131.Referenced from the NCCN Clinical Practice Guidelines in Oncology (NCCN Guidelines®) for Antiemesis V.2.2025. ©National Comprehensive Cancer Network, Inc, 2025. All rights reserved. Available online: https://www.nccn.org/guidelines/guidelines-detail?category=3&id=1415 (accessed on 3 December 2025). To view the most recent and complete version of the guideline, go online to NCCN.org. NCCN makes no warranties of any kind whatsoever regarding their content, use or application and disclaims any responsibility for their application or use in any way.

[132] Notini G., Naldini M.M., Sica L., Viale G., Rognone A., Zambelli S., Zucchinelli P., Piras M., Bosi C., Mariani M. (2024). Management of trastuzumab deruxtecan-related nausea and vomiting in real-world practice. Front. Oncol..

[133] Sakai H., Tsurutani J., Ozaki Y., Ishiguro H., Nozawa K., Yamanaka T., Aogi K., Matsumoto K., Iwasa T., Tokiwa M. (2024). A randomized, double-blind, placebo-controlled phase II study of olanzapine based prophylactic antiemetic therapy for delayed and persistent nausea and vomiting in patients with HER2-positive or HER2-low breast cancer treated with trastuzumab deruxtecan: ERICA study (WJOG14320B). Ann. Oncol..

[134] Bianchini G., Park Y.H., Rugo H.S., Licata L., Iihara H., Massagrande M., Dato F., Scotté F., Jordan K., Aapro M.S. (2024). Perceptions of antibody drug conjugate (ADC)-induced nausea and vomiting: Results of a survey of healthcare providers at ESMO. ESMO Breast Cancer.

[135] Zuo S., Cheng H., Wang Z., Liu T., Chen S., Tian L., Lin L. (2023). Nonpharmacological interventions for cancer-related fatigue: A literature review. Asia Pac. J. Oncol. Nurs..

[136] US National Library of Medicine Scalp Cooling in MBC. https://clinicaltrials.gov/study/NCT04986579.

[137] Kruse M., Abraham J. (2018). Management of chemotherapy-induced alopecia with scalp cooling. J. Oncol. Pract..

[138] Repetto L. (2003). Greater risks of chemotherapy toxicity in elderly patients with cancer. J. Support. Oncol..

[139] Evans N., Anton A., Wong R., Lok S.W., De Boer R., Malik L., Greenberg S., Yeo B., Nott L., Richardson G. (2021). Abstract PS6-35: Real world outcomes in elderly women with HER2 positive advanced breast cancer. Cancer Res..

[140] Krop I.E., Wildiers H., Hurvitz S.A., Cortes J., Im S.-A., Iwata H., Andre F., Saura C., Modi S., Kim S.-B. (2023). An age-specific pooled analysis of trastuzumab deruxtecan (T-DXd) in patients (pts) with HER2-positive (HER2+) metastatic breast cancer (mBC) from DESTINY-Breast01, -02, and -03. J. Clin. Oncol..

[141] Modi S., Salgado R., Guarneri V., Alagappan D., Dennis N., Newell A.H., Boran A., Morsli O., Llombart-Cussac A. (2024). Abstract PO2-19-06: An open-label, interventional, multicenter study of trastuzumab deruxtecan monotherapy in patients with unresectable and/or metastatic HER2-low or HER2 immunohistochemistry 0 breast cancer: DESTINY-Breast15. Cancer Res..

[142] Moore K.N., Sabanathan D., Du Y., Duan H., Li X., Wang F., Marathe O., Yang H., Makker V., Growdon W. (2023). Safety and efficacy of DB-1303 in patients with advanced/metastatic solid tumors: A multicenter, open-label, first-in-human, phase 1/2a study. J. Clin. Oncol..

[143] Bardia A., O’Sullivan C.C., Mahtani R.L., Blau S., Abdulla N.E., Ali A., Bansal R., Meyer J.M., George M.A., Han H.S. (2024). An open-label, multicenter, phase 2 study to evaluate the safety and efficacy of BB-1701, a novel antibody drug conjugate (ADC) targeting human epidermal growth factor receptor 2 (HER2), in previously treated patients with HER2-positive (HER2+) or HER2-low unresectable or metastatic breast cancer (BC). J. Clin. Oncol..

[144] Lewis G.D., Li G., Guo J., Yu S.F., Fields C.T., Lee G., Zhang D., Dragovich P.S., Pillow T., Wei B. (2024). The HER2-directed antibody-drug conjugate DHES0815A in advanced and/or metastatic breast cancer: Preclinical characterization and phase 1 trial results. Nat. Commun..

[145] Wang J., Liu Y., Zhang Q., Li W., Feng J., Wang X., Fang J., Han Y., Xu B. (2024). Disitamab vedotin, a HER2-directed antibody-drug conjugate, in patients with HER2-overexpression and HER2-low advanced breast cancer: A phase I/Ib study. Cancer Commun..

[146] Banerji U., van Herpen C.M.L., Saura C., Thistlethwaite F., Lord S., Moreno V., Macpherson I.R., Boni V., Rolfo C., de Vries E.G.E. (2019). Trastuzumab duocarmazine in locally advanced and metastatic solid tumours and HER2-expressing breast cancer: A phase 1 dose-escalation and dose-expansion study. Lancet Oncol..

[147] US National Library of Medicine Phase II Open-Label Study of ARX788 (Anti-HER2 Antibody Drug Conjugate (ADC)) for Patients with HER2-Low Locally Advanced Unresectable or Metastatic Breast Cancer. https://clinicaltrials.gov/study/NCT06224673.

[148] Dent R.A., Cescon D.W., Bachelot T., Jung K.H., Shao Z.M., Saji S., Traina T.A., Vukovic P., Mapiye D., Maxwell M.J. (2023). TROPION-Breast02: Datopotamab deruxtecan for locally recurrent inoperable or metastatic triple-negative breast cancer. Future Oncol..

[149] Schmid P., Wysocki P.J., Ma C.X., Park Y.H., Fernandes R., Lord S., Baird R.D., Prady C., Jung K.H., Asselah J. (2023). 379MO Datopotamab deruxtecan (Dato-DXd) + durvalumab (D) as first-line (1L) treatment for unresectable locally advanced/metastatic triple-negative breast cancer (a/mTNBC): Updated results from BEGONIA, a phase Ib/II study. Ann. Oncol..

[150] Bardia A., Pusztai L., Albain K., Ciruelos E.M., Im S.A., Hershman D., Kalinsky K., Isaacs C., Loirat D., Testa L. (2024). TROPION-Breast03: A randomized phase III global trial of datopotamab deruxtecan ± durvalumab in patients with triple-negative breast cancer and residual invasive disease at surgical resection after neoadjuvant therapy. Ther. Adv. Med. Oncol..

[151] McArthur H.L., Tolaney S.M., Dent R., Schmid P., Asselah J., Liu Q., Meisel J.L., Niikura N., Park Y.H., Werutsky G. (2025). TROPION-Breast04: A randomized phase III study of neoadjuvant datopotamab deruxtecan (Dato-DXd) plus durvalumab followed by adjuvant durvalumab versus standard of care in patients with treatment-naïve early-stage triple negative or HR-low/HER2- breast cancer. Ther. Adv. Med. Oncol..

[152] Schmid P., Oliveira M., O’Shaughnessy J., Cristofanilli M., Graff S.L., Im S.A., Loi S., Saji S., Wang S., Cescon D.W. (2025). TROPION-Breast05: A randomized phase III study of Dato-DXd with or without durvalumab versus chemotherapy plus pembrolizumab in patients with PD-L1-high locally recurrent inoperable or metastatic triple-negative breast cancer. Ther. Adv. Med. Oncol..

[153] Rugo H., Cortés J., Curigliano G., Barrios C., Punie K., Park Y., Iwata H., Young A., Ren X., Cinar P. (2024). ASCENT-07: A phase 3, randomized, open-label study of sacituzumab govitecan versus treatment of physician’s choice in patients with HR+/HER2– inoperable, locally advanced, or metastatic breast cancer post-endocrine therapy. Cancer Res..

[154] Vaz Batista M., Pérez-García J.M., Garrigós L., García-Sáenz J., Cortez P., Racca F., Blanch S., Ruiz-Borrego M., Fernández-Ortega A., Fernández-Abad M. (2025). The DEBBRAH trial: Trastuzumab deruxtecan in HER2-positive and HER2-low breast cancer patients with leptomeningeal carcinomatosis. Med.

[155] Jhaveri K., André F., Hamilton E., Schmid P., Anders C., Testa L., Ganshina I., Lu Y.-S., Im S.-A., Young R. (2024). Trastuzumab deruxtecan (T-DXd) in combination with anastrozole or fulvestrant in patients with HER2-low HR+ advanced/metastatic breast cancer: A Phase 1b, open-label, multicenter, dose-expansion study (DESTINY-Breast08). Cancer Res..

[156] US National Library of Medicine Trastuzumab Deruxtecan (T-DXd) in Patients Who Have Hormone Receptor-Negative and Hormone Receptor-Positive HER2-Low or HER2 IHC 0 Metastatic Breast Cancer. https://clinicaltrials.gov/study/NCT05950945.

[157] Guarneri V., Passos Coelho J.L., Duhoux F.P., Egle D., García-Sáenz J., Penault-Llorca F., Selander K., Wildiers H., Zaman K., Laeis P. (2024). Study design for DESTINY-Breast Respond HER2-low Europe: T-DXd in patients with HER2-low advanced breast cancer. Future Oncol..

[158] US National Library of Medicine A Randomized Phase II Study to Evaluate the Safety and Efficacy of Trastuzumab Deruxtecan Versus CDK4/6 Inhibitor-Based Endocrine Therapy as First-Line Therapy of HR-Positive and HER2-Low/Ultralow Advanced Breast Cancer Patients Classified as Non-Luminal Subtype According to Gene Expression Profiling. https://clinicaltrials.gov/study/NCT06486883.

[159] US National Library of Medicine T-DXd Therapy for HER2-Low Breast Cancer Patients with Brain Metastases (TUXEDO-4). https://clinicaltrials.gov/study/NCT06048718.

[160] Marhold M., Vaz Batista M., Blancas I., Morales C., Saura-Manich C., Saavedra C., Ruíz-Borrego M., Cortez P., Slebe F., Campolier M. (2025). TUXEDO-4: Phase II study of trastuzumab-deruxtecan in HER2-low breast cancer with new or progressing brain metastases. Future Oncol..

[161] US National Library of Medicine A phase II Non-Comparative Trial of Datopotamab Deruxtecan (Dato-DXd) or Trastuzumab Deruxtecan (T-DXd) in Patients with Metastatic HER2-Low Breast Cancer After Progression on Prior Antibody Drug Conjugate therapy. https://clinicaltrials.gov/study/NCT06533826.

